# Supplementary material for: Multi-species host range of staphylococcal phages isolated from wastewater
Source: Nat Commun. 2021 Nov 29;12:6965. doi: 10.1038/s41467-021-27037-6 (PMC8629997; doi:10.1038/s41467-021-27037-6)
Supplement: Supplementary file 1 — Supplementary information [file 41467_2021_27037_MOESM1_ESM.pdf]

Supplementary Information

Multi-species host range of staphylococcal phages isolated from wastewater

Pauline C. Göller[1], Tabea Elsener[1], Dominic Lorgé[1], Natasa Radulovic[1], Viona Bernardi[1], Annika Naumann[1], Nesrine Amri[1], Ekaterina Khatchatourova[1], Felipe Hernandes Coutinho[2], Martin J. Loessner[1], Elena Gómez-Sanz[1,3]*

[1]Institute of Food, Nutrition and Health, ETH Zurich, 8092 Zurich, Switzerland.

[2]Área de Microbiología Molecular, Centro de Investigación Biomédica de La Rioja (CIBIR), Logroño, Spain.

[3] Departamento de Producción Vegetal y Microbiología, Universidad Miguel Hernández, San Juan de Alicante, Spain.

*Correspondance: elena.gomez@hest.ethz.ch; elenagomez.titus@gmail.com

Supplementary Figure 1

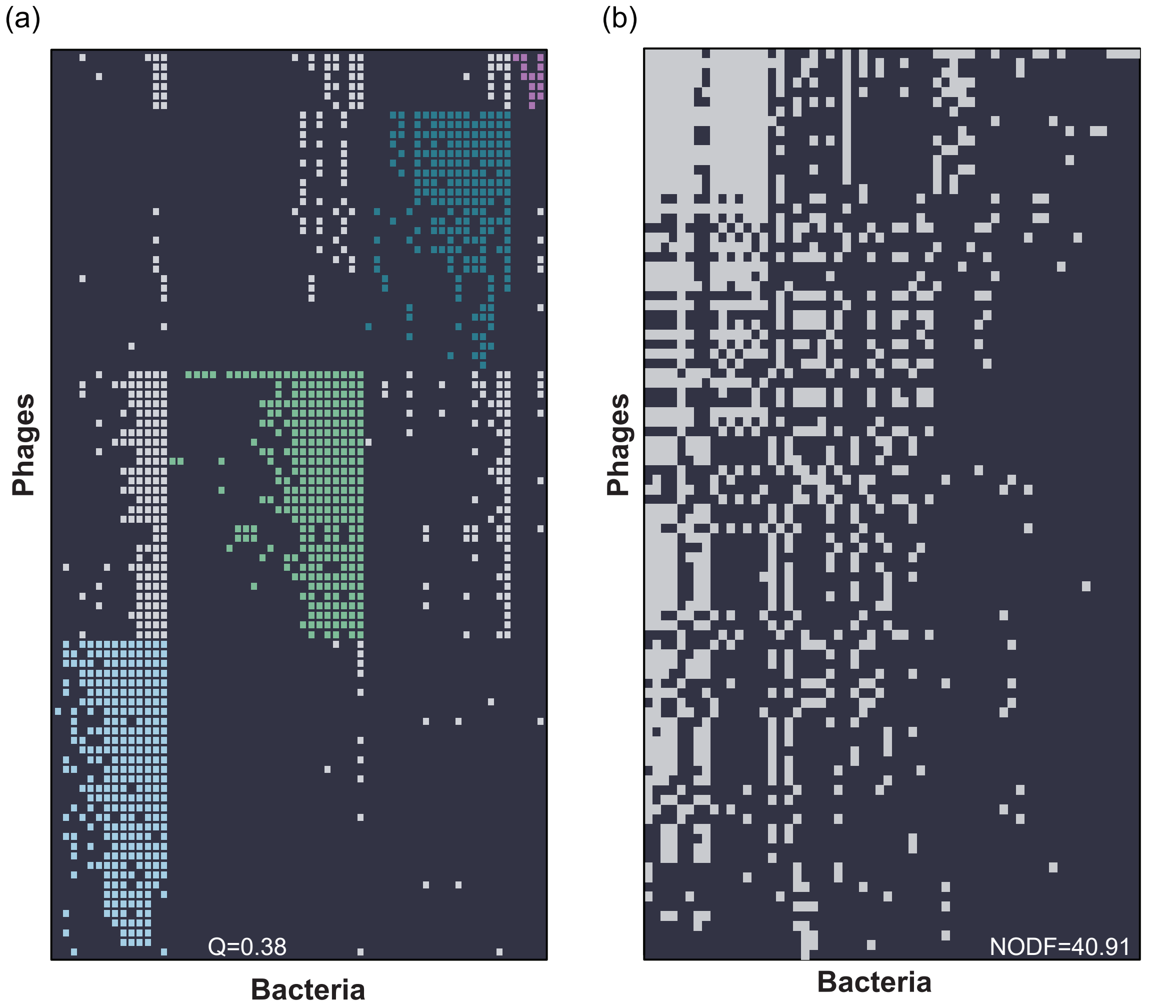

Matrix representation of the modular and nested network structure. The matrix is composed of 60 phage permissive staphylococcal strains from 27 species and 94 phages. The rows represent bacteria, and columns represent phages. Grey cells illustrate reported infections. (a) Illustration of the modular sorting. Infections within modules are represented in color. The modularity level (Q), estimated with the lpbrim package in R, is indicated on the bottom line. (b) Illustration of the nestedness sorting. The matrix is now arranged to maximize nestedness. The nestedness, estimated with the NODF function, is indicated in the lower right corner. Both algorithms are described and explained in[11]. Source data are provided as a Source Data file.

Supplementary Figure 2

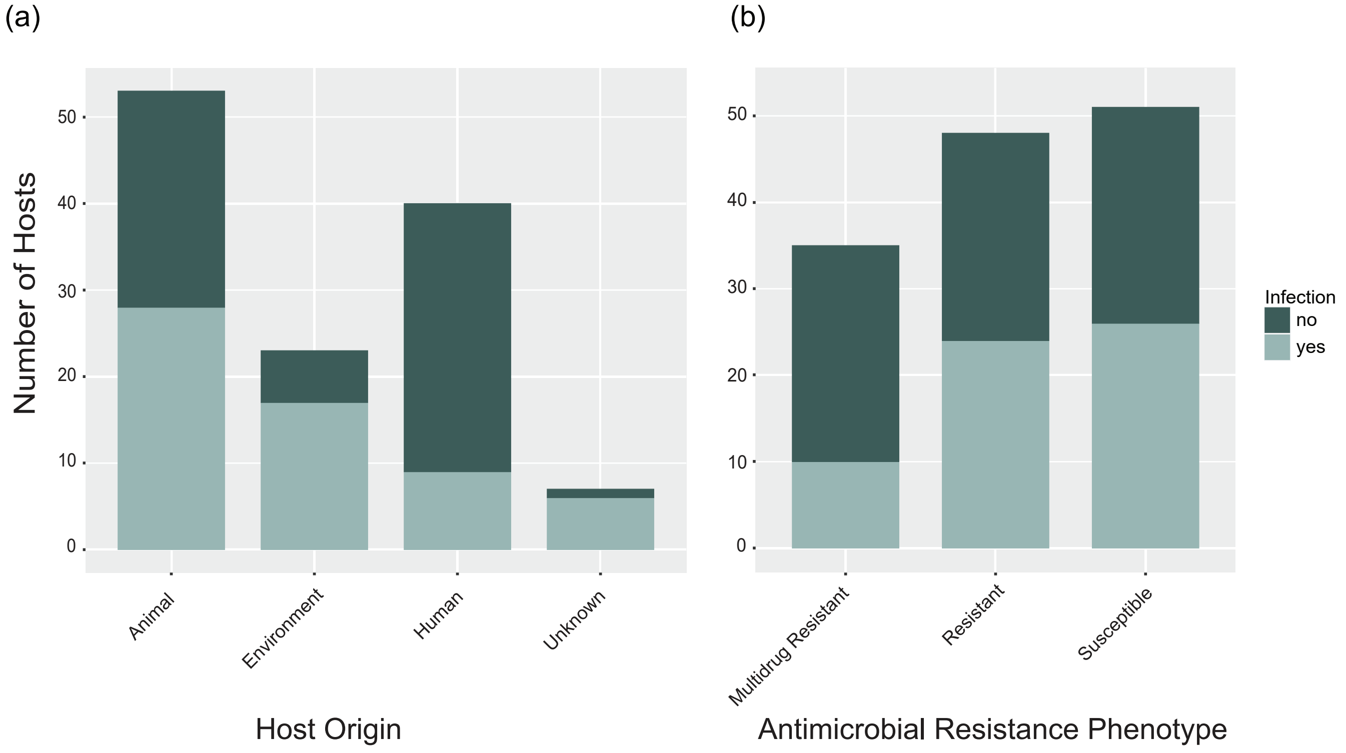

Host susceptibility towards phage infection. Depicted are the number of phage resistant and permissive hosts that are clustered according to their (a) isolation origin and (b) antimicrobial resistant phenotype. Source data are provided as a Source Data file.

Supplementary Figure 3

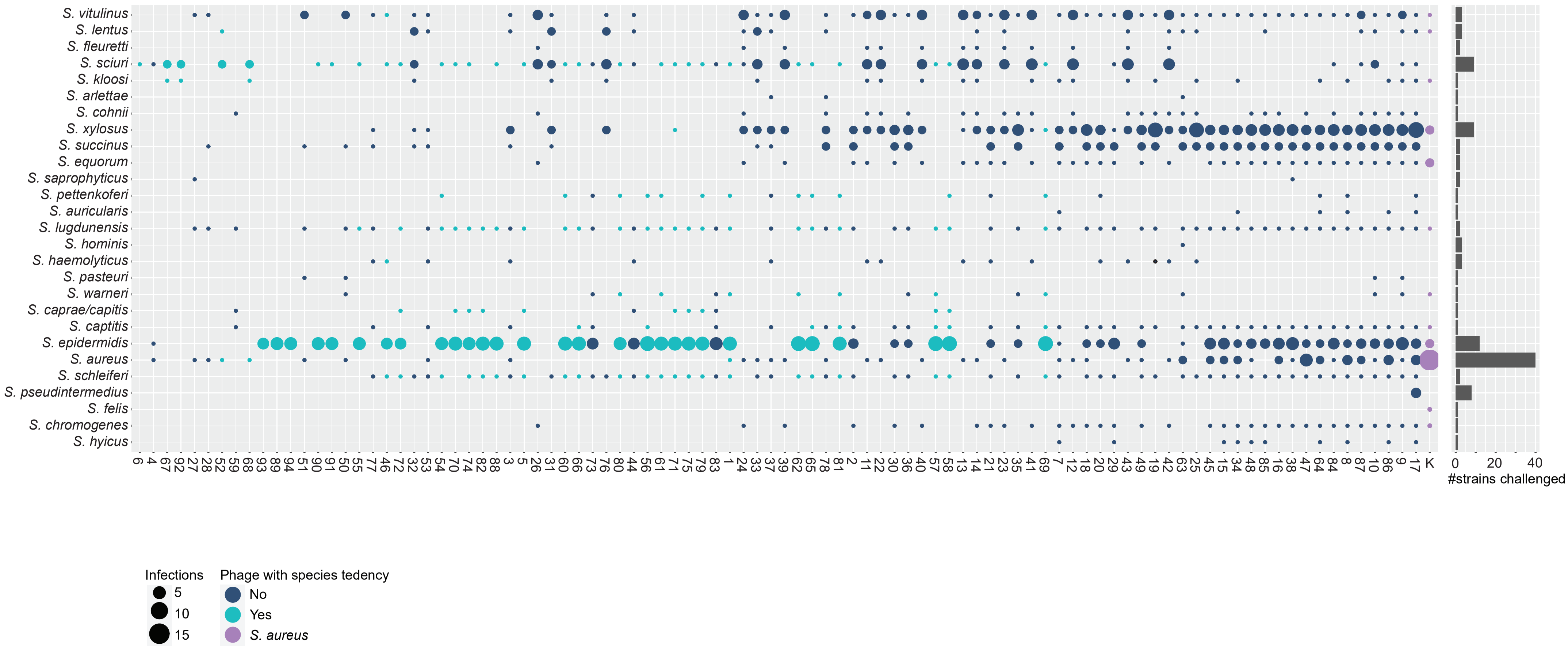

Illustration of the host ranges collapsed on the species level for all isolated phages. Phages on the x-axis are sorted from narrow (left) to broad host range (right). Species on the y-axis are sorted after phylogenetic relationship in species groups[13]. A phage host range is depicted as a column, where infection of a staphylococcal species is illustrated using circles. For each respective species, the area of the circle is scaled according to the number of strains a phage can replicate on (scale: 1-15). The total number of strains challenged per species is depicted in the bar-chart on the right. Host ranges on this host array are colored as follows: Phage with species tendency (≥ 50 % of all infections on a single species) in turquoise; phages with no species tendency in dark blue; polyvalent phage K in violet. Phages are abbreviated with their final unique numerical identifier (PG-2021_*). Source data are provided as a Source Data file.

Supplementary Figure 4

(a)

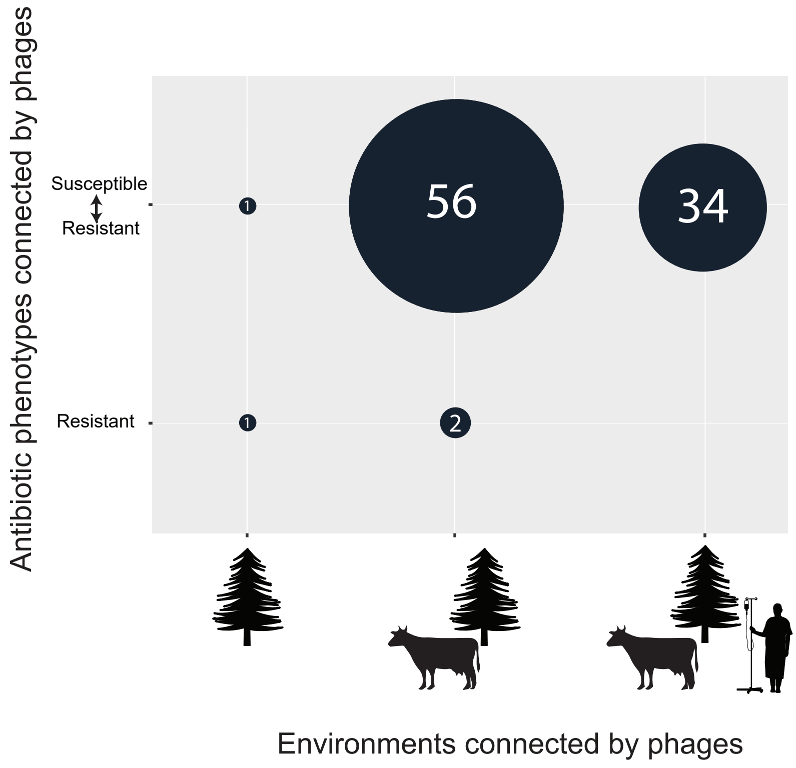

(b)

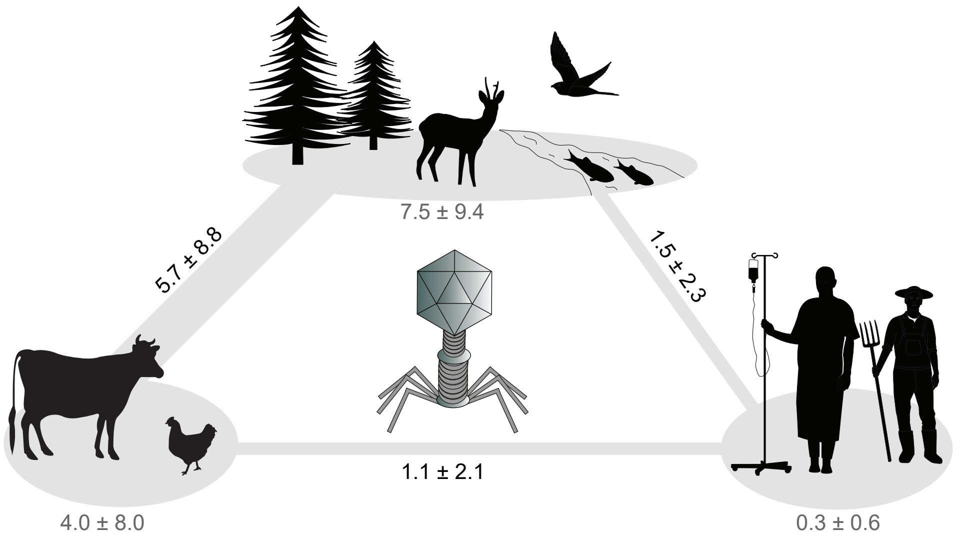

Phages connecting hosts of different antimicrobial resistant phenotypes and epidemiologic backgrounds. (a) Phages infecting either exclusively drug resistant, or drug resistant and susceptible bacteria are represented as rows. The number of phages connecting hosts from the environmental ecosystem, both the environmental and veterinary ecosystem, or all three ecosystems are represented as columns. (b) The average number of shared phages (mean±sd) between hosts within or across an ecosystem is depicted. Staphylococcal hosts are classified according to their isolation origin into environmental, veterinary, or human associated strains. Source data are provided as a Source Data file.

Supplementary Figure 5

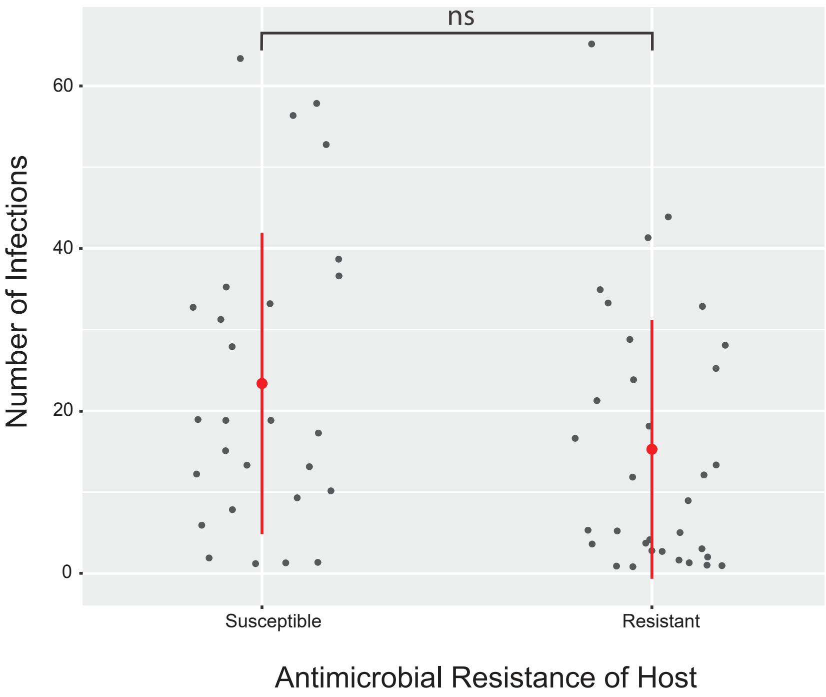

Phage susceptibility of strains classified after drug resistance phenotypes. For each strain, the number of total phages infecting this host was determined and depicted on the y-axis. On the x-axis, strains are classified according to their antimicrobial resistance phenotype. The average number of phages infecting a host phenotype is illustrated in red (mean±sd). There is no significant difference in the infection of antimicrobial susceptible or resistant hosts (Two-sided Wilcoxon rank sum test with continuity correction, W = 568.5, n = 60, p-value = 0.068). Source data are provided as a Source Data file.

Supplementary Figure 6

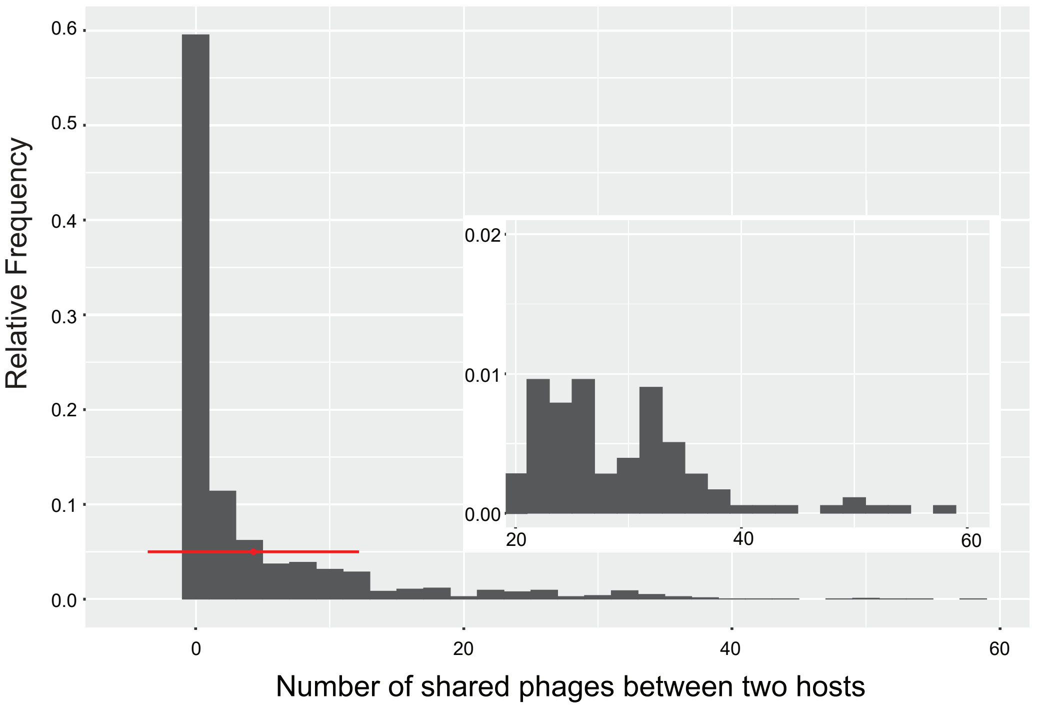

The number of shared phages between hosts in the bipartite network projection. With our natural phage community being present, two bacterial hosts share between zero and 58 phages. The relative frequency indicates how many bacterial hosts share the respective number of phages (bin width two). On average, each host pair is connected by 4.2 ± 7.9 (n=1770) different phages (mean ± sd in red). Source data are provided as a Source Data file.

Supplementary Figure 7

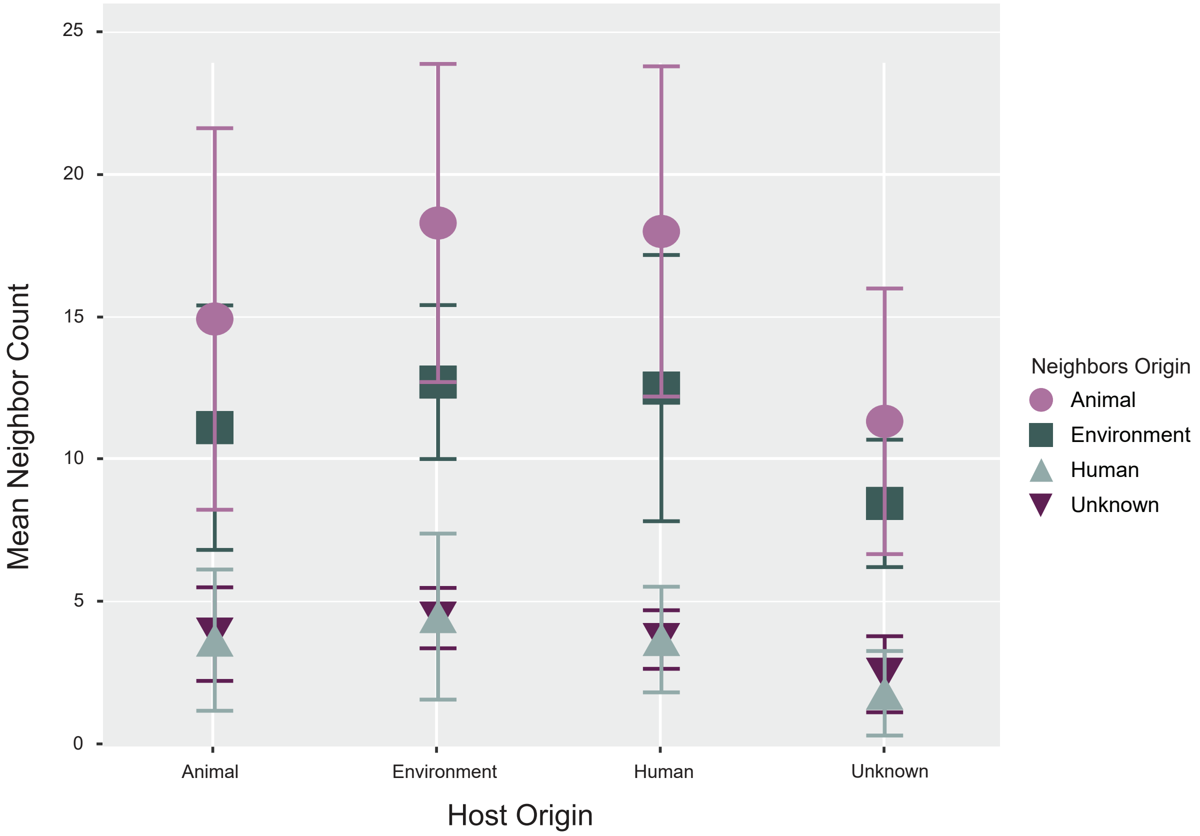

Mean neighbor count of phage permissive hosts in the bipartite network projection. Phage permissive hosts (n = 60) were categorized after their isolation origin, and the number of direct neighbors connected through phages was counted. Neighbors themselves were subdivided according to their isolation origin. A total of 2030 datapoints were generated, which correspond to 1030 edges analyzed in both directions (2 X 1030). For each single strain, the number of neighbors from a specific environment was calculated and then averaged over 2030.On average, hosts isolated from animals revealed to have 33.5 ± 14.5 (n = 28) neighbors, environmental hosts 39.9 ± 11.6 (n = 17), hosts isolated from the human biome 24 ± 9 (n = 9), and hosts of unknown isolation origin 37.8 ± 12.6 (n = 6) neighbors. The data are expressed as mean ± sd. Source data are provided as a Source Data file.

Supplementary Table 1

Summary of the constitution and phage isolation efficiency for each enrichment cocktail.

Cocktail	Strains Origin	# Strains	# Isolated Phages
A	Randomly combined	9	14
B	Animal	9	32
C	WWTP/surface water	8	54
D	Labstrains	11	26
E	WWTP/surface water	9	29

Supplementary Table 2

The number of isolated phages for enrichment species, and their corresponding number of successful and unsuccessful enrichment strains.

Enrichment Species	# Enrichment Strains	# of successfull Enrichment Strains	# Enriched Phages	Enriched Phages (%)
S. aureus	14	2	10	6.45%
S. caprae/capitis	1	1	9	5.81%
S. chromogenes	2	1	4	2.58%
S. devriesei	1	0	0	0.00%
S. epidermidis	6	5	56	36.13%
S. equorum	1	1	3	1.94%
S. fleuretti	1	1	1	0.65%
S. haemolyticus	3	1	4	2.58%
S. lentus	1	1	6	3.87%
S. pseudintermedius	1	1	1	0.65%
S. saprophyticus	1	1	1	0.65%
S. schleiferi	1	1	8	5.16%
S. sciuri	4	3	21	13.55%
S. simulans	1	0	0	0.00%
S. succinus	2	2	9	5.81%
S. vitulinus	3	3	19	12.26%
S. xylosus	3	2	3	1.94%
		26	155	

Supplementary Table 3

Compilation of isolation hosts for all induced phages, and their respective efficiency.

Species	Strain	# Isolated Phages
S. epidermidis	C3910	2
S. epidermidis	C6869	5
S. epidermidis	I0515	6
S. epidermidis	I0564	4
S. epidermidis	NCC100655	5
S. sciuri	C6888	2

Supplementary Table 4

Summary of all phage isolation and discrimination advances on each staphylococcal species.

Species	# Successfull Isolation Strains	# Isolated phages	# Different phages
S. aureus	2	10	5
S. caprae/capitis	1	9	1
S. chromogenes	1	4	1
S. epidermidis	6	78	23
S. equorum	1	3	1
S. fleuretti	1	1	0
S. haemolyticus	1	4	3
S. lentus	1	6	3
S. pseudintermedius	1	1	1
S. saprophyticus	1	1	1
S. schleiferi	1	8	5
S. sciuri	3	23	8
S. succinus	2	9	7
S. vitulinus	3	19	14
S. xylosus	2	3	3
		179	76

Supplementary Table 5

Taxonomic diversity of species and strains detected in each module after modularity sorting of the phage-bacteria interaction matrix.

Module	Species Groups	Species	Number of Strains
1	Hyicus-Intermedius	*S. schleiferi*	1
1	Epidermidis-Aureus	*S. caprae/capitis*	1
1	Epidermidis-Aureus	*S. epidermidis*	7
1	Epidermidis-Aureus	*S. lugdunensis*	1
1	Epidermidis-Aureus	*S. warneri*	1
1	Saprophyticus	*S. nepalensis*	1
1	Saprophyticus	*S. pettenkoferi*	1
1	Sciuri	***S. sciuri***[1]	1
2	Epidermidis-Aureus	***S. aureus***	1
2	Epidermidis-Aureus	***S. haemolyticus***	1
2	Epidermidis-Aureus	*S. pasteuri*	1
2	Saprophyticus	*S. cohnii*	1
2	Saprophyticus	*S. kloosii*	1
2	Saprophyticus	*S. saprophyticus*	1
2	Saprophyticus	***S. xylosus***	1
2	Sciuri	*S. fleuretti*	1
2	Sciuri	***S. lentus***	2
2	Sciuri	***S. sciuri***	5
2	Sciuri	*S. vitulinus*	3
3	Epidermidis-Aureus	***S. haemolyticus***	1
3	Epidermidis-Aureus	*S. hominis*	1
3	Saprophyticus	*S. arlettae*	1
3	Sciuri	***S. lentus***	1
4	Hyicus-Intermedius	*S. chromogenes*	1
4	Hyicus-Intermedius	*S. hyicus*	1
4	Hyicus-Intermedius	*S. pseudintermedius*	3
4	Epidermidis-Aureus	***S. aureus***	6
4	Epidermidis-Aureus	*S. capitis*	1
4	Auricularis	*S. auricularis*	1
4	Saprophyticus	*S. equorum*	1
4	Saprophyticus	*S. succinus*	2
4	Saprophyticus	***S. xylosus***	8

[1] species in bold occur in one than 1 module

Supplementary Table 6

Primer sequences.

Primer Name	Primer Sequence 5'-3'
catpC221-fw	GTAACAATAGCAGCTTTTTATTGCCT
catpC221-rv	TAAATAATGAAGCATGGTAACCATCAC
catpC221-probe	AGCATGATGAAGCTGTAAGGCAACTGGTAT
