# Supplementary material for: Multi-species host range of staphylococcal phages isolated from wastewater
Source: Nat Commun. 2021 Nov 29;12:6965. doi: 10.1038/s41467-021-27037-6 (PMC8629997; doi:10.1038/s41467-021-27037-6)
Supplement: Supplementary file 3 — Description of Additional Supplementary Files [file 41467_2021_27037_MOESM3_ESM.pdf]

Description of Additional Supplementary Files

File Name: Supplementary Data 1
Description: Bacterial host selected for the enrichment cocktail constitution and phage isolation.

File Name: Supplementary Data 2
Description: Compilation of all enrichment hosts and their respective efficiency in phage isolation.

File Name: Supplementary Data 3
Description: Compilation over all isolated phages, their isolation origin and hosts, as well as the corresponding enrichment cocktail. Phages with equal cluster numbers (column F) had identical host ranges on 123 different bacteria.

File Name: Supplementary Data 4
Description: Biadjacency matrix of the phage-bacterium network with characteristics and phenotypes of all challenged bacteria.

File Name: Supplementary Data 5
Description: Corresponding enrichment cocktail for each phage cluster.

File Name: Supplementary Data 6
Description: Isolation frequency and hosts for all isolated phages.

File Name: Supplementary Data 7
Description: Biadjacency matrix of the phage-bacterium network with characteristics and phenotypes of all isolated phages, including phage K.

File Name: Supplementary Data 8
Description: Strain abundance and phage permissiveness for each bacterial species included in the host array.

File Name: Supplementary Data 9
Description: Host range characteristics for all 94 staphylococcal phages concerning infection of strain from different ecosystems and antimicrobial resistance phenotypes.

File Name: Supplementary Data 10
Description: Rendered R markdowns.
