# Supplementary material for: Multi-species host range of staphylococcal phages isolated from wastewater
Source: Nat Commun. 2021 Nov 29;12:6965. doi: 10.1038/s41467-021-27037-6 (PMC8629997; doi:10.1038/s41467-021-27037-6)
Supplement: Supplementary file 13 — Supplementary Data 10 [file 41467_2021_27037_MOESM13_ESM.pdf]

Multi-species host range of staphylococcal phages isolated from wastewater

Pauline C. Göller[1], Tabea Elsener[1], Dominic Lorgé[1], Natasa Radulovic[1], Viona Bernardi[1], Annika Naumann[1], Nesrine Amri[1], Ekaterina Khatchatourova[1], Felipe Hernandes Coutinho[2], Martin J. Loessner[1], Elena Gómez-Sanz[1,3]*

[1]Institute of Food, Nutrition and Health, ETH Zurich, 8092 Zurich, Switzerland.

[2]Área de Microbiología Molecular, Centro de Investigación Biomédica de La Rioja (CIBIR), Logroño, Spain.

[3] Departamento de Producción Vegetal y Microbiología, Universidad Miguel Hernández, San Juan de Alicante, Spain.

*Correspondance: elena.gomez@hest.ethz.ch; elenagomez.titus@gmail.com

Rendered R Markdowns for

(i) Network Analysis (page 2 - 50)

(ii) Modularity and Nestedness (page 52 - 54)

(iii) Species Specificity (page 56 - 57)

HR Network Analysis

Contents

Packages **2**

Data preparation **3**
- Host range matrix . . . 3
- Host range species level . . . 4
- Host information . . . 4

General Network descriptives: Network 123x94 **10**
- Hosts . . . 10
  - Number of CoPS / CoNS strains . . . 10
  - Phage susceptible CoPS/CoNS strains . . . 11
    - CoPS . . . 11
    - CoNS . . . 11
  - Number of host species . . . 11
  - Infections on the strain level . . . 11
  - Infections on the species level . . . 12
  - Resistant / susceptible . . . 12
    - Infections on resistant / susceptible hosts . . . 12
  - Source . . . 12
    - Infections of animal/human/environmental hosts . . . 13
- Phages . . . 13
  - Maximal number of infections . . . 14
  - Average number of infections . . . 14
  - Phage infecting only one single host . . . 14
  - Phages infecting a single species . . . 14
  - Infections on species groups . . . 14
- Network initiation . . . 16
- Degree . . . 16
  - Phages . . . 16
  - Hosts . . . 17
  - Node degree by species . . . 17
    - Node degree by resistance pattern . . . 18
    - Node degree by source . . . 18

Network 60x94 **18**
- Hosts . . . 19
  - Species . . . 19
  - CoPS / CoNS . . . 19
  - Resistance phenotype . . . 19
  - Origin . . . 19
  - Infections / host . . . 20
- Phages . . . 20
  - Successful infections / phage . . . 20
  - Phages infecting resistant hosts . . . 20

Network initialization . . . 21
Degree . . . 21
Whole network . . . 21
Phages . . . 21
Hosts . . . 23
Node degree by coagulase type . . . 26
CoNS . . . 26
CoPS . . . 26
Node degree by species . . . 26
Node degree by resistance pattern . . . 27
Node degree by source . . . 28
Network diameter . . . 28
Correlation between resistance phenotype and number of phage infections . . . 28

Bipartite projections **30**
Functions . . . 30
Host network . . . 30
Diameter host network . . . 31
Connectance . . . 31
Degree . . . 31
Degree by species . . . 31
Degree by resistance pattern . . . 32
Degree by source . . . 32
Neighbours . . . 32
Neighbors of a particular species . . . 33
Neighbours of a specific environment . . . 35
Neighbours of a specific resistance phenotype . . . 37
Shared phages . . . 40
Average number of connecting phages . . . 41
Between species . . . 41
Shared phages between environments . . . 43
Shared phages between resistance phenotypes . . . 45
Shared phages between cluster groups . . . 47
Shared phages between specific hosts . . . 49

Network analysis:

Dataset: 123 Phages, 94 Phages (Phage K removed)

Host attributes: Source (Animal/Environment/Human), Resistance Phenotype (susceptible/resistant), Species (n = 27), Cluster group, Species Group, Coagulase Type (COPS/CONS/NaN) note that mdr hosts are included in the resistant group

General Network Descriptives: - Degree - Connectance - Phages connecting 2 Hosts - Number of Neighbours of a specific Attribute

Packages

```
sessionInfo()
```

```
## R version 3.6.2 (2019-12-12)
## Platform: x86_64-apple-darwin15.6.0 (64-bit)
## Running under: macOS  10.16
##
## Matrix products: default
```

```
## BLAS:   /Library/Frameworks/R.framework/Versions/3.6/Resources/lib/libRblas.0.dylib
## LAPACK: /Library/Frameworks/R.framework/Versions/3.6/Resources/lib/libRlapack.dylib
##
## locale:
## [1] de_CH.UTF-8/de_CH.UTF-8/de_CH.UTF-8/C/de_CH.UTF-8/de_CH.UTF-8
##
## attached base packages:
## [1] stats     graphics  grDevices utils     datasets  methods   base
##
## other attached packages:
##  [1] ggpubr_0.2.5    magrittr_1.5    janitor_2.0.1   igraph_1.2.5
##  [5] forcats_0.5.0   stringr_1.4.0   dplyr_0.8.5     purrr_0.3.3
##  [9] readr_1.3.1     tidyr_1.0.2     tibble_2.1.3    ggplot2_3.3.0
## [13] tidyverse_1.3.0
##
## loaded via a namespace (and not attached):
##  [1] tidyselect_1.0.0 xfun_0.12        haven_2.2.0      lattice_0.20-40
##  [5] snakecase_0.11.0 colorspace_1.4-1 vctrs_0.2.4      generics_0.0.2
##  [9] htmltools_0.4.0  yaml_2.2.1       rlang_0.4.5      pillar_1.4.3
## [13] glue_1.3.2       withr_2.1.2      DBI_1.1.0        dbplyr_1.4.2
## [17] modelr_0.1.6     readxl_1.3.1     lifecycle_0.2.0  ggsignif_0.6.0
## [21] munsell_0.5.0    gtable_0.3.0     cellranger_1.1.0 rvest_0.3.5
## [25] evaluate_0.14    knitr_1.28       fansi_0.4.1      broom_0.5.5
## [29] Rcpp_1.0.4       scales_1.1.1     backports_1.1.5  jsonlite_1.6.1
## [33] fs_1.3.2         hms_0.5.3        digest_0.6.25    stringi_1.4.6
## [37] grid_3.6.2       cli_2.0.2        tools_3.6.2      crayon_1.3.4
## [41] pkgconfig_2.0.3  xml2_1.2.5       reprex_0.3.0     lubridate_1.7.4
## [45] assertthat_0.2.1 rmarkdown_2.1    httr_1.4.1       rstudioapi_0.11
## [49] R6_2.4.1         nlme_3.1-145     compiler_3.6.2
```

Data preparation

Phage HR: incidence matrix of hosts(x-Axis, 124) and phages(y-Axis, 94) 1: infection/interaction 0: no infection Phage K: reference, is removed for the network analysis

Host range matrix

```r
# import data: Phage HR
Phage_HR <- read.csv("Data/Phage_HR_binary_woL.csv", header=FALSE,
                     row.names=1, sep=";", stringsAsFactors=FALSE)

# remove whitespace (not uniform colnames in the 2 datasets)
Phage_HR[1,67] <- "JW4341"
Phage_HR[1,68] <- "JW2311"

# set Phages as rownames
Phage_HR <- row_to_names(Phage_HR, row_number = 1)

# remove Phage K and Listeria from dataset
Phage_HR <- Phage_HR[which(rownames(Phage_HR)!= "K"),]

# save information about coagulase type and species
Host_info <- Phage_HR[95:96,] %>% t() %>% as.data.frame()
```

```r
Host_info <- mutate(Host_info, Strain = rownames(Host_info))

COPS <- Host_info[Host_info$Coagulase == "COPS",] %>% select(Strain) %>% as.list()
CONS <- Host_info[Host_info$Coagulase == "CONS",] %>% select(Strain) %>% as.list()

# get rid of species and coagulase rows
Phage_HR <- Phage_HR[1:94,]
#dim(Phage_HR)
#tail(Phage_HR)

# turn it into a numeric matrix
rn <- rownames(Phage_HR)
HR <- Phage_HR %>% mutate_all(function(x) as.numeric(as.character(x))) %>% as.matrix()
rownames(HR) <- rn

phages <- rownames(HR)
hosts <-colnames(HR)
dim(HR) # dimensions of the matrix: 94 phages and 123 bacterial hosts
```

```
## [1]  94 123
```

```r
#View(HR)
#colnames(HR)
```

```r
sum(HR) # number of infections
```

```
## [1] 1135
```

Host range species level

Host range on the species level, values can be greater than one when multiple strains of a species are infected. HR_binary: only 0/1 matrix where 1 indicates that at least one strain of the respective species is infected by the phage

```r
HR_Species <- read.csv("Data/Phage_HR_Species_newn.csv", sep=";", row.names = 1)

# remove phage K and Listeria
HR_Species <- HR_Species[which(rownames(HR_Species)!= "K"),]
HR_Species <- HR_Species[,which(colnames(HR_Species)!= "L..ivanovii")]

# turn into numeric matrix
rn <- rownames(HR_Species)
HR_Species <- HR_Species %>% mutate_all(function(x) as.numeric(as.character(x))) %>%
  as.matrix()
rownames(HR_Species) <- rn

# make the matrix binary,
# 0: not infected at all, 1: at least one strain of the species is infected by the phage
HR_Species_binary <- as.matrix((HR_Species > 0) + 0)
dim(HR_Species_binary) # 94 phages, 32 strains
```

```
## [1] 94 32
```

Host information

Data frame which contains all relevant information about the 123 hosts:

- Species
- Origin/Source
- Resistance Phenotype: note that MDR hosts are included in the resistant group
- Cluster Group
- Species Group
- Coagulase Type

```
All_hosts <- read.csv("Data/All_hosts_newn.csv", sep=";") %>% select(c('Strain', 'Source',
                                                                         'MDR'))
#head(All_hosts)
attributes_hosts <- unique(All_hosts)

attributes_hosts$Strain <- as.character(attributes_hosts$Strain)
attributes_hosts[which(attributes_hosts$Strain == "KM1345 "), "Strain"] <- "KM 1345 "
attributes_hosts[which(attributes_hosts$Strain == "KM1352 "), "Strain"] <- "KM 1352 "
attributes_hosts[which(attributes_hosts$Strain == "IMD0819 "), "Strain"] <- "IMD 0819 "
#dim(attributes_hosts)
#head(attributes_hosts)

Host_info <- left_join(Host_info, attributes_hosts, by = "Strain")
Host_info$Strain <- as.character(Host_info$Strain)
#Host_info[Host_info$Strain =="C8946 ", "Strain"] <- "C8946"

# we do not distinguish between mdr and r

# change header
colnames(Host_info)[5] <- "Resistance"

# include mdr into Resistance
Host_info[Host_info$Resistance == "MDR", "Resistance"] <- "Resistant"

# change susceptible to uppercase
Host_info$Resistance <- as.character(Host_info$Resistance)
Host_info[Host_info$Resistance == "susceptible", "Resistance"] <- "Susceptible"

# change levels
Host_info$Resistance <- as.factor(Host_info$Resistance)
#levels(Host_info$Resistance)
#dim(Host_info)

#cluster groups
species_groups <- read.csv("Data/Staph_Genus_sorted_123.csv",header=TRUE,
                           stringsAsFactors = F, sep = ";")
colnames(species_groups) <- c("Strain", "Species", "Cluster", "Species_Group")
species_groups <- species_groups[,c("Strain", "Cluster", "Species_Group")]
Host_info <- left_join(Host_info, species_groups, by= "Strain")
#dim(Host_info)
Host_info
```

```
##                 Species Coagulase     Strain      Source  Resistance
## 1           S. arlettae      CONS     I4042       Animal   Resistant
## 2        S. auricularis      CONS    I2040B       Animal Susceptible
## 3            S. capitis      CONS     C6866 Environment   Resistant
## 4     S. caprae/capitis      CONS     I0510       Animal Susceptible
## 5        S. chromogenes      CONS     C5799 Environment Susceptible
```

```
## 6             S. cohnii     CONS      I2468      Animal   Resistant
## 7        S. epidermidis     CONS  NCC100655     Unknown Susceptible
## 8        S. epidermidis     CONS    DSM1798     Unknown   Resistant
## 9        S. epidermidis     CONS       S414     Unknown Susceptible
## 10       S. epidermidis     CONS      C6869 Environment   Resistant
## 11       S. epidermidis     CONS      I0564      Animal Susceptible
## 12       S. epidermidis     CONS      I0515      Animal Susceptible
## 13       S. epidermidis     CONS      C2825       Human   Resistant
## 14       S. epidermidis     CONS      C3945       Human   Resistant
## 15       S. epidermidis     CONS      C3910       Human   Resistant
## 16       S. epidermidis     CONS      C3041       Human   Resistant
## 17       S. epidermidis     CONS      C2817       Human   Resistant
## 18       S. epidermidis     CONS      C2826       Human   Resistant
## 19           S. equorum     CONS      C5824 Environment Susceptible
## 20           S. equorum     CONS      I3667      Animal Susceptible
## 21             S. felis     CONS      I1365      Animal Susceptible
## 22         S. fleuretti     CONS      C5825 Environment   Resistant
## 23         S. fleuretti     CONS      JW205      Animal   Resistant
## 24      S. haemolyticus     CONS      C6858 Environment   Resistant
## 25      S. haemolyticus     CONS      I2994      Animal Susceptible
## 26      S. haemolyticus     CONS      C2709       Human   Resistant
## 27           S. hominis     CONS      I0042      Animal Susceptible
## 28           S. hominis     CONS      C5862 Environment   Resistant
## 29           S. hominis     CONS      C5835 Environment Susceptible
## 30            S. hyicus     CONS       MP01      Animal Susceptible
## 31           S. kloosii     CONS      I2998      Animal   Resistant
## 32            S. lentus     CONS      C5847 Environment Susceptible
## 33            S. lentus     CONS      C3986      Animal   Resistant
## 34            S. lentus     CONS      C3031       Human   Resistant
## 35       S. lugdunensis     CONS      I4003      Animal Susceptible
## 36       S. lugdunensis     CONS     I0507       Animal   Resistant
## 37        S. nepalensis     CONS      I0838      Animal   Resistant
## 38         S. pasteuri     CONS      I1462      Animal   Resistant
## 39      S. pettenkoferi     CONS      I0029      Animal Susceptible
## 40     S. saprophyticus     CONS      C6887 Environment   Resistant
## 41     S. saprophyticus     CONS      I2004      Animal   Resistant
## 42        S. schleiferi     CONS      I0032      Animal Susceptible
## 43            S. sciuri     CONS      C6888 Environment   Resistant
## 44            S. sciuri     CONS      C6854 Environment   Resistant
## 45            S. sciuri     CONS      C5868 Environment   Resistant
## 46            S. sciuri     CONS M1687-1/10       Human   Resistant
## 47            S. sciuri     CONS      C2866      Animal   Resistant
## 48            S. sciuri     CONS    M4488/09      Animal   Resistant
## 49            S. sciuri     CONS    M1215/10      Animal   Resistant
## 50            S. sciuri     CONS      I2907      Animal   Resistant
## 51            S. sciuri     CONS      C2865      Animal   Resistant
## 52          S. simulans     CONS   DSM20322       Human   Resistant
## 53          S. simulans     CONS      C5844 Environment Susceptible
## 54          S. succinus     CONS      C6852 Environment Susceptible
## 55          S. succinus     CONS      I4644      Animal Susceptible
## 56         S. vitulinus     CONS      C6867 Environment Susceptible
## 57         S. vitulinus     CONS      C5817 Environment Susceptible
## 58         S. vitulinus     CONS      C5821 Environment Susceptible
## 59           S. warneri     CONS      C5816 Environment Susceptible
```

```
## 60          S. xylosus      CONS      C5863 Environment   Resistant
## 61          S. xylosus      CONS  DSM20266        Human Susceptible
## 62          S. xylosus      CONS       MP05       Animal Susceptible
## 63          S. xylosus      CONS M4212-2/09       Human   Resistant
## 64          S. xylosus      CONS      C5806 Environment   Resistant
## 65          S. xylosus      CONS M1997-2/10      Animal   Resistant
## 66          S. xylosus      CONS      I2595      Animal   Resistant
## 67          S. xylosus      CONS     JW4341      Animal   Resistant
## 68          S. xylosus      CONS     JW2311      Animal   Resistant
## 69           S. aureus      COPS         3A     Unknown Susceptible
## 70           S. aureus      COPS      Twort     Unknown Susceptible
## 71           S. aureus      COPS      C2448       Human   Resistant
## 72           S. aureus      COPS      C1569       Human   Resistant
## 73           S. aureus      COPS      C7697      Animal   Resistant
## 74           S. aureus      COPS      C1891      Animal   Resistant
## 75           S. aureus      COPS     RN4220     Unknown Susceptible
## 76           S. aureus      COPS     C5515        Human   Resistant
## 77           S. aureus      COPS      C3972       Human Susceptible
## 78           S. aureus      COPS      C3968       Human Susceptible
## 79           S. aureus      COPS      C2438       Human   Resistant
## 80           S. aureus      COPS      C2435       Human   Resistant
## 81           S. aureus      COPS      C2444       Human   Resistant
## 82           S. aureus      COPS      C3012       Human   Resistant
## 83           S. aureus      COPS      C3931       Human   Resistant
## 84           S. aureus      COPS      C2920       Human   Resistant
## 85           S. aureus      COPS      C3494       Human   Resistant
## 86           S. aureus      COPS      C1795      Animal   Resistant
## 87           S. aureus      COPS     C6595       Animal   Resistant
## 88           S. aureus      COPS      C5650      Animal   Resistant
## 89           S. aureus      COPS      C3883      Animal   Resistant
## 90           S. aureus      COPS      C7925      Animal   Resistant
## 91           S. aureus      COPS      C5611      Animal   Resistant
## 92           S. aureus      COPS      C2355       Human   Resistant
## 93           S. aureus      COPS      C1890      Animal   Resistant
## 94           S. aureus      COPS      C1532      Animal   Resistant
## 95           S. aureus      COPS      C6828       Human   Resistant
## 96           S. aureus      COPS      C6319       Human   Resistant
## 97           S. aureus      COPS      C5429       Human   Resistant
## 98           S. aureus      COPS      C5036       Human   Resistant
## 99           S. aureus      COPS      C5033       Human   Resistant
## 100          S. aureus      COPS      C5029       Human   Resistant
## 101          S. aureus      COPS      C4684       Human   Resistant
## 102          S. aureus      COPS      C4680       Human   Resistant
## 103          S. aureus      COPS      C3544       Human   Resistant
## 104          S. aureus      COPS      C2881       Human   Resistant
## 105          S. aureus      COPS      C6865 Environment Susceptible
## 106          S. aureus      COPS      C3912       Human   Resistant
## 107          S. aureus      COPS      PS187       Human Susceptible
## 108 S. pseudintermedius      COPS      2638A     Unknown Susceptible
## 109 S. pseudintermedius      COPS      C5829 Environment   Resistant
## 110 S. pseudintermedius      COPS      C3930      Animal   Resistant
## 111 S. pseudintermedius      COPS      C2915       Human   Resistant
## 112 S. pseudintermedius      COPS      C3874      Animal Susceptible
## 113 S. pseudintermedius      COPS      C5329      Animal   Resistant
```

```
## 114 S. pseudintermedius      COPS      C5352      Animal   Resistant
## 115 S. pseudintermedius      COPS      C3870      Animal   Resistant
## 116 S. pseudintermedius      COPS      C5347      Animal   Resistant
## 117       S. schleiferi      COPS      I3823      Animal Susceptible
## 118         E. faecalis       NaN     C3735        Human   Resistant
## 119         E. faecalis       NaN     C8946        Human   Resistant
## 120            M. canis       NaN   KM 1345       Animal Susceptible
## 121            M. canis       NaN KM 450/13       Animal   Resistant
## 122     M. caseolyticus       NaN   KM 1352       Animal Susceptible
## 123     M. caseolyticus       NaN  IMD 0819       Animal   Resistant
##                        Cluster      Species_Group
## 1          Arlettae-kloosii      Saprophyticus
## 2               Auricularis        Auricularis
## 3               Epidermidis Epidermidis-Aureus
## 4               Epidermidis Epidermidis-Aureus
## 5                    Hyicus Hyicus-Intermedius
## 6         Cohnii-nepalensis      Saprophyticus
## 7               Epidermidis Epidermidis-Aureus
## 8               Epidermidis Epidermidis-Aureus
## 9               Epidermidis Epidermidis-Aureus
## 10              Epidermidis Epidermidis-Aureus
## 11              Epidermidis Epidermidis-Aureus
## 12              Epidermidis Epidermidis-Aureus
## 13              Epidermidis Epidermidis-Aureus
## 14              Epidermidis Epidermidis-Aureus
## 15              Epidermidis Epidermidis-Aureus
## 16              Epidermidis Epidermidis-Aureus
## 17              Epidermidis Epidermidis-Aureus
## 18              Epidermidis Epidermidis-Aureus
## 19            Saprophyticus      Saprophyticus
## 20            Saprophyticus      Saprophyticus
## 21                   Hyicus Hyicus-Intermedius
## 22                   Sciuri             Sciuri
## 23                     <NA>               <NA>
## 24             Haemolyticus Epidermidis-Aureus
## 25             Haemolyticus Epidermidis-Aureus
## 26             Haemolyticus Epidermidis-Aureus
## 27             Haemolyticus Epidermidis-Aureus
## 28             Haemolyticus Epidermidis-Aureus
## 29             Haemolyticus Epidermidis-Aureus
## 30                   Hyicus Hyicus-Intermedius
## 31         Arlettae-kloosii      Saprophyticus
## 32                   Sciuri             Sciuri
## 33                   Sciuri             Sciuri
## 34                   Sciuri             Sciuri
## 35              Lugdunensis Epidermidis-Aureus
## 36                     <NA>               <NA>
## 37        Cohnii-nepalensis      Saprophyticus
## 38                  Warneri Epidermidis-Aureus
## 39  Pettenkoferi-Massiliensis      Saprophyticus
## 40            Saprophyticus      Saprophyticus
## 41            Saprophyticus      Saprophyticus
## 42              Intermedius Hyicus-Intermedius
## 43                   Sciuri             Sciuri
```

```
## 44                   Sciuri             Sciuri
## 45                   Sciuri             Sciuri
## 46                     <NA>               <NA>
## 47                   Sciuri             Sciuri
## 48                     <NA>               <NA>
## 49                     <NA>               <NA>
## 50                   Sciuri             Sciuri
## 51                   Sciuri             Sciuri
## 52       Simulans-carnosus           Simulans
## 53       Simulans-carnosus           Simulans
## 54           Saprophyticus      Saprophyticus
## 55           Saprophyticus      Saprophyticus
## 56                   Sciuri             Sciuri
## 57                   Sciuri             Sciuri
## 58                   Sciuri             Sciuri
## 59                  Warneri Epidermidis-Aureus
## 60           Saprophyticus      Saprophyticus
## 61                     <NA>               <NA>
## 62           Saprophyticus      Saprophyticus
## 63                     <NA>               <NA>
## 64           Saprophyticus      Saprophyticus
## 65                     <NA>               <NA>
## 66           Saprophyticus      Saprophyticus
## 67                     <NA>               <NA>
## 68                     <NA>               <NA>
## 69                     <NA>               <NA>
## 70                S. aureus Epidermidis-Aureus
## 71                S. aureus Epidermidis-Aureus
## 72                S. aureus Epidermidis-Aureus
## 73                S. aureus Epidermidis-Aureus
## 74                S. aureus Epidermidis-Aureus
## 75                S. aureus Epidermidis-Aureus
## 76                     <NA>               <NA>
## 77                S. aureus Epidermidis-Aureus
## 78                S. aureus Epidermidis-Aureus
## 79                S. aureus Epidermidis-Aureus
## 80                S. aureus Epidermidis-Aureus
## 81                S. aureus Epidermidis-Aureus
## 82                S. aureus Epidermidis-Aureus
## 83                S. aureus Epidermidis-Aureus
## 84                S. aureus Epidermidis-Aureus
## 85                S. aureus Epidermidis-Aureus
## 86                S. aureus Epidermidis-Aureus
## 87                     <NA>               <NA>
## 88                S. aureus Epidermidis-Aureus
## 89                S. aureus Epidermidis-Aureus
## 90                S. aureus Epidermidis-Aureus
## 91                S. aureus Epidermidis-Aureus
## 92                S. aureus Epidermidis-Aureus
## 93                S. aureus Epidermidis-Aureus
## 94                S. aureus Epidermidis-Aureus
## 95                S. aureus Epidermidis-Aureus
## 96                S. aureus Epidermidis-Aureus
## 97                S. aureus Epidermidis-Aureus
```

```
## 98                  S. aureus Epidermidis-Aureus
## 99                  S. aureus Epidermidis-Aureus
## 100                 S. aureus Epidermidis-Aureus
## 101                 S. aureus Epidermidis-Aureus
## 102                 S. aureus Epidermidis-Aureus
## 103                 S. aureus Epidermidis-Aureus
## 104                 S. aureus Epidermidis-Aureus
## 105                 S. aureus Epidermidis-Aureus
## 106                 S. aureus Epidermidis-Aureus
## 107                 S. aureus Epidermidis-Aureus
## 108               Intermedius Hyicus-Intermedius
## 109               Intermedius Hyicus-Intermedius
## 110               Intermedius Hyicus-Intermedius
## 111               Intermedius Hyicus-Intermedius
## 112               Intermedius Hyicus-Intermedius
## 113               Intermedius Hyicus-Intermedius
## 114               Intermedius Hyicus-Intermedius
## 115               Intermedius Hyicus-Intermedius
## 116               Intermedius Hyicus-Intermedius
## 117               Intermedius Hyicus-Intermedius
## 118                      <NA>               <NA>
## 119                      <NA>               <NA>
## 120                      <NA>               <NA>
## 121                      <NA>               <NA>
## 122                      <NA>               <NA>
## 123                      <NA>               <NA>
```

General Network descriptives: Network 123x94

```r
# number of Phages(rows) and Hosts(columns)
dim(HR)
```

```
## [1]  94 123
```

```r
# number of interactions(infections)
sum(HR)
```

```
## [1] 1135
```

Hosts

Number of CoPS / CoNS strains

```r
# number of CoPS
length(COPS$Strain)
```

```
## [1] 49
```

```r
# number of CoNS
length(CONS$Strain)
```

```
## [1] 68
```

```r
# number of staphylococcal hosts
length(COPS$Strain) + length(CONS$Strain)
```

```
## [1] 117
```

Phage susceptible CoPS/CoNS strains

CoPS

```r
# CoPS that are infected by the phage community
sum(HR[,colnames(HR) %in% COPS$Strain] %>% colSums() != 0 )
```

```
## [1] 11
```

```r
# percent of infected CoPS
sum(HR[,colnames(HR) %in% COPS$Strain] %>% colSums() != 0 ) *100 /sum(colSums(HR) != 0)
```

```
## [1] 18.33333
```

```r
#percent of infections on CoPS
HR[,colnames(HR) %in% COPS$Strain] %>% colSums() %>% sum()*100/sum(colSums((HR)))
```

```
## [1] 11.10132
```

CoNS

```r
# CONS infected by the phage community
sum(HR[,colnames(HR) %in% CONS$Strain] %>% colSums() != 0 )
```

```
## [1] 49
```

```r
# percent of infected CONS
sum(HR[,colnames(HR) %in% CONS$Strain] %>% colSums() != 0 ) *100 /sum(colSums(HR) != 0)
```

```
## [1] 81.66667
```

```r
#percent of infections on COPS
HR[,colnames(HR) %in% CONS$Strain] %>% colSums() %>% sum()*100/sum(colSums((HR)))
```

```
## [1] 88.89868
```

Number of host species

```r
length(unique(Host_info$Species))
```

```
## [1] 32
```

Infections on the strain level

```r
# summary on the number of infections on single hosts
colSums(HR) %>% summary()
```

```
##    Min. 1st Qu.  Median    Mean 3rd Qu.    Max.
##   0.000   0.000   0.000   9.228  13.000  65.000
```

```r
# standard deviation
colSums(HR) %>% sd()
```

```
## [1] 15.42715
```

```r
# n
length(colSums(HR))
```

```
## [1] 123
```

```
# strain with most infections
colSums(HR)[colSums(HR) == max(colSums(HR))]
```

```
## I0507
##    65
```

Infections on the species level

```
# summary on the number of infections a species
colSums(HR_Species_binary) %>% summary()
```

```
##    Min. 1st Qu.  Median    Mean 3rd Qu.    Max.
##    0.00    1.75   15.50   22.72   40.75   65.00
```

```
# standard deviation
colSums(HR_Species_binary) %>% sd()
```

```
## [1] 22.22645
```

```
# n
length(colSums(HR_Species_binary))
```

```
## [1] 32
```

Resistant / susceptible

```
#number of resistant hosts
Host_info[Host_info$Resistance == "Resistant", 1] %>% length()
```

```
## [1] 84
```

```
#number of susceptible hosts
Host_info[Host_info$Resistance == "Susceptible", 1] %>% length()
```

```
## [1] 39
```

Infections on resistant / susceptible hosts

```
# all resistant hosts
resistant_hosts <- Host_info[Host_info$Resistance == 'Resistant', "Strain"]
# number of infections on resistant hosts
HR[,colnames(HR) %in% resistant_hosts] %>% sum()
```

```
## [1] 505
```

```
# all susceptible hosts
susceptible_hosts <- Host_info[Host_info$Resistance == 'Susceptible', "Strain"]
# number of infections on susceptible hosts
HR[,colnames(HR) %in% susceptible_hosts] %>% sum()
```

```
## [1] 630
```

Source

animal/human/environment

```
# animal
Host_info[Host_info$Source == "Animal", "Strain"] %>% length()
```

```
## [1] 53
```

```r
# human
Host_info[Host_info$Source == "Human", "Strain"] %>% length()
```

```
## [1] 40
```

```r
# environment
Host_info[Host_info$Source == "Environment", "Strain"] %>% length()
```

```
## [1] 23
```

```r
# unknown
Host_info[Host_info$Source == "Unknown", "Strain"] %>% length()
```

```
## [1] 7
```

Infections of animal/human/environmental hosts

```r
# animal hosts
animal_hosts <- Host_info[Host_info$Source == 'Animal', "Strain"]
HR[,colnames(HR) %in% animal_hosts] %>% sum()
```

```
## [1] 515
```

```r
# human hosts
human_hosts <- Host_info[Host_info$Source == 'Human', "Strain"]
HR[,colnames(HR) %in% human_hosts] %>% sum()
```

```
## [1] 41
```

```r
# environmental hosts
environmental_hosts <- Host_info[Host_info$Source == 'Environment', "Strain"]
HR[,colnames(HR) %in% environmental_hosts] %>% sum()
```

```
## [1] 418
```

```r
# unknown source hosts
na_hosts <- Host_info[Host_info$Source == 'Unknown', "Strain"]
HR[,colnames(HR) %in% na_hosts] %>% sum()
```

```
## [1] 161
```

Phages

```r
# average and max number of infections (strains)
summary(rowSums(HR))
```

```
##    Min. 1st Qu.  Median    Mean 3rd Qu.    Max.
##    1.00    9.00   12.00   12.07   15.00   32.00
```

```r
# standard deviation
sd(rowSums(HR))
```

```
## [1] 5.354607
```

```r
# n
length(rowSums(HR))
```

```
## [1] 94
```

```r
# phage that infects most Hosts
names(rowSums(HR))[rowSums(HR) == max(rowSums(HR))]
```

```
## [1] "PG-2021_17"
```

```r
# number of infections
rowSums(HR)[rowSums(HR) == max(rowSums(HR))]
```

```
## PG-2021_17
##         32
```

Maximal number of infections

```r
# number of species infected
max(rowSums(HR_Species_binary))
```

```
## [1] 18
```

```r
# which phage
names(rowSums(HR_Species_binary))[rowSums(HR_Species_binary) ==
                                    max(rowSums(HR_Species_binary))]
```

```
## [1] "PG-2021_17"
```

Average number of infections

```r
#mean
mean(rowSums(HR_Species_binary))
```

```
## [1] 7.734043
```

```r
#sd
sd(rowSums(HR_Species_binary))
```

```
## [1] 3.663759
```

```r
# n
length(rowSums(HR_Species_binary))#n
```

```
## [1] 94
```

Phage infecting only one single host

```r
names(rowSums(HR))[rowSums(HR) == 1]
```

```
## [1] "PG-2021_6"
```

Phages infecting a single species

```r
names(rowSums(HR_Species_binary))[rowSums(HR_Species_binary) == 1]
```

```
## [1] "PG-2021_6"  "PG-2021_89" "PG-2021_93" "PG-2021_94"
```

Infections on species groups

```r
#count for each phage how many species groups it infects
species_groups_infected <- data.frame("Phage", "number_of_species_groups_infected",
```

```r
                                          stringsAsFactors = FALSE)
for (phage in rownames(HR)){
  #print(phage)
  sg <- c()
  for (host in colnames(HR)){
    h<- HR[rownames(HR)==phage, host]
    if (h == 1){
      #print(host)
      sg_host <- as.character(Host_info[Host_info$Strain==host, "Species_Group"])
      #print(sg_host)
      sg <- append(sg, sg_host)
    }
  }
  species_groups_infected <-rbind(species_groups_infected, c(phage, length(unique(sg))))
}

species_groups_infected <- row_to_names(species_groups_infected, 1)

#how many species groups in total?
#length(unique(Host_info$Species_Group))
```

```r
#phages that infect all species groups
sum(species_groups_infected$number_of_species_groups_infected ==
      length(unique(Host_info$Species_Group)))
```

```
## [1] 0
```

```r
#no phage infects all species_groups
```

```r
#max number of species groups infected
species_groups_infected[species_groups_infected$number_of_species_groups_infected ==
                        max(species_groups_infected$number_of_species_groups_infected),]
```

```
##          Phage number_of_species_groups_infected
## 8    PG-2021_7                                 6
## 9    PG-2021_8                                 6
## 18 PG-2021_17                                  6
## 35 PG-2021_34                                  6
## 65 PG-2021_64                                  6
## 87 PG-2021_86                                  6
```

```r
#How many phages connect >1 species group?
sum(species_groups_infected$number_of_species_groups_infected > 1 )
```

```
## [1] 90
```

```r
#how many different specis groups are on average infected by a phage?
#mean
mean(as.numeric(as.character(species_groups_infected$number_of_species_groups_infected)))
```

```
## [1] 4.37234
```

```r
#sd
sd(as.numeric(as.character(species_groups_infected$number_of_species_groups_infected)))
```

```
## [1] 1.135773
```

Network initiation

```
# initialize network
net <- graph_from_incidence_matrix(HR)

# add source, resistance_phenotype, number of resistances and species group as vertex attributes
for (i in 1:length(Host_info$Strain)){
  #print(Host_info$Strain[i])
  vertex_attr(net, 'Source', index = as.character(Host_info$Strain[i]) ) <-
    as.character(Host_info$Source[i])
  vertex_attr(net, 'Resistance_phenotype', index = as.character(Host_info$Strain[i]) ) <-
    as.character(Host_info$MDR[i])
  vertex_attr(net, 'number_resistances', index = as.character(Host_info$Strain[i]) ) <-
    Host_info$Number_of_r[i]
  vertex_attr(net, 'Species', index = as.character(Host_info$Strain[i]) ) <-
    as.character(Host_info$Species[i])
  vertex_attr(net, 'Species_Group', index = as.character(Host_info$Strain[i]) ) <-
    as.character(Host_info$Species_Group[i])
}
#get.vertex.attribute(net)
```

Degree

```
# overall degree
mean(degree(net, mode="all"))
```

```
## [1] 10.46083
```

```
#sd
sd(degree(net, mode = "all"))
```

```
## [1] 12.19705
```

```
#n
length(V(net))
```

```
## [1] 217
```

Phages

```
# mean degree phages
mean(degree(net)[names(degree(net)) %in% phages])
```

```
## [1] 12.07447
```

```
#sd
sd(degree(net)[names(degree(net)) %in% phages])
```

```
## [1] 5.354607
```

```
#n
length(phages)
```

```
## [1] 94
```

Hosts

```
# mean degree hosts
mean(degree(net)[names(degree(net)) %in% hosts])
```

```
## [1] 9.227642
```

```
#sd
sd(degree(net)[names(degree(net)) %in% hosts])
```

```
## [1] 15.42715
```

```
#n
length(hosts)
```

```
## [1] 123
```

Node degree by species

```
species_info <- data.frame("Species", "n", "mean_degree", "sd_degree",
                           stringsAsFactors = F)
species <- unique(Host_info$Species)
for (s in species) {
  hosts <- as.character(Host_info[Host_info$Species == s, "Strain"])
  species_info <- rbind(species_info, c(s, length(hosts), mean(degree(net, hosts)),
                                        sd(degree(net, hosts))))
  }
species_info <- row_to_names(species_info,1)
species_info
```

```
##                Species  n       mean_degree         sd_degree
## 2          S. arlettae  1                 3              <NA>
## 3       S. auricularis  1                 6              <NA>
## 4           S. capitis  1                44              <NA>
## 5    S. caprae/capitis  1                13              <NA>
## 6       S. chromogenes  1                33              <NA>
## 7            S. cohnii  1                29              <NA>
## 8       S. epidermidis 12             21.25  22.7880550847468
## 9           S. equorum  2              17.5  24.7487373415292
## 10            S. felis  1                 0              <NA>
## 11        S. fleuretti  2               6.5  9.19238815542512
## 12     S. haemolyticus  3  5.33333333333333  6.11010092660779
## 13          S. hominis  3 0.333333333333333 0.577350269189626
## 14           S. hyicus  1                10              <NA>
## 15          S. kloosii  1                24              <NA>
## 16           S. lentus  3  8.66666666666667  7.23417813807024
## 17      S. lugdunensis  2              32.5  45.9619407771256
## 18       S. nepalensis  1                 1              <NA>
## 19         S. pasteuri  1                 4              <NA>
## 20     S. pettenkoferi  1                19              <NA>
## 21    S. saprophyticus  2                 1   1.4142135623731
## 22       S. schleiferi  2              31.5  44.5477272147525
## 23           S. sciuri  9  10.6666666666667  10.7470926301023
## 24         S. simulans  2                 0                 0
## 25         S. succinus  2                35  2.82842712474619
## 26        S. vitulinus  3  28.3333333333333  21.5715862498179
```

```
## 27          S. warneri  1                15              <NA>
## 28          S. xylosus  9                16  14.4395290781937
## 29           S. aureus 39  1.53846153846154  5.43790862394994
## 30 S. pseudintermedius  9 0.333333333333333               0.5
## 31         E. faecalis  2                 0                 0
## 32            M. canis  2                 0                 0
## 33     M. caseolyticus  2                 0                 0
```

Node degree by resistance pattern

```
resistance_phenotype_info <- data.frame("Resistance phenotype", "n", "mean_degree",
                                        "sd_degree",  stringsAsFactors = F)
res_pattern <- unique(Host_info$Resistance)
for (r in res_pattern) {
  hosts <- as.character(Host_info[Host_info$Resistance == r, "Strain"])
  resistance_phenotype_info <- rbind(resistance_phenotype_info,
                                     c(r, length(hosts), mean(degree(net, hosts)),
                                       sd(degree(net, hosts))))
  }
resistance_phenotype_info <- row_to_names(resistance_phenotype_info,1)
resistance_phenotype_info
```

```
##   Resistance phenotype  n      mean_degree        sd_degree
## 2            Resistant 84 6.01190476190476 12.4140360199281
## 3          Susceptible 39 16.1538461538462 18.8519696884332
```

Node degree by source

```
source_info <- data.frame("Source", "n", "mean_degree", "sd_degree",
                          stringsAsFactors = F)
source <- unique(Host_info$Source)
for (s in source) {
  hosts <- as.character(Host_info[Host_info$Source == s, "Strain"])
  source_info <- rbind(source_info,
                       c(s, length(hosts), mean(degree(net, hosts)),
                         sd(degree(net, hosts))))
  }
source_info <- row_to_names(source_info,1)
source_info
```

```
##        Source  n      mean_degree        sd_degree
## 2      Animal 53 9.71698113207547 16.4776974662147
## 3 Environment 23 18.1739130434783  17.020448060316
## 4     Unknown  7               23 20.0997512422418
## 5       Human 40            1.025 2.51648411632029
```

Network 60x94

```
# remove all hosts that haven't been infected at all

HR_60 <- HR[,colSums(HR) != 0] # only include the hosts that are infected
#dim(HR_60) # 94 Phages, 60 Hosts
#sum(HR_60) # total number of interactions
hosts_60 <-colnames(HR_60)
```

```r
#head(HR)
Host_info_60 <- Host_info %>% filter(Strain %in% hosts_60)
```

Hosts

Species

```r
# host species
Host_info %>% filter(Strain %in% hosts_60) %>% select(Species) %>% unique() %>% as.list()
```

```
## $Species
##  [1] S. arlettae         S. auricularis      S. capitis
##  [4] S. caprae/capitis   S. chromogenes      S. cohnii
##  [7] S. epidermidis      S. equorum          S. fleuretti
## [10] S. haemolyticus     S. hominis          S. hyicus
## [13] S. kloosii          S. lentus           S. lugdunensis
## [16] S. nepalensis       S. pasteuri         S. pettenkoferi
## [19] S. saprophyticus    S. sciuri           S. succinus
## [22] S. vitulinus        S. warneri          S. xylosus
## [25] S. aureus           S. pseudintermedius S. schleiferi
## 32 Levels: E. faecalis M. canis M. caseolyticus S. arlettae ... S. xylosus
```

CoPS / CoNS

```r
# CoPS strains
COPS <- Host_info %>% filter(Strain %in% hosts_60) %>%  filter(Coagulase == "COPS")
length(COPS[,2])
```

```
## [1] 11
```

```r
# CoNS strains
CONS <- Host_info %>% filter(Strain %in% hosts_60) %>%  filter(Coagulase == "CONS")
length(CONS[,2])
```

```
## [1] 49
```

Resistance phenotype

```r
#resistant hosts
length(Host_info_60[Host_info_60$Resistance == "Resistant", "Strain"])
```

```
## [1] 34
```

```r
#Susceptible
length(Host_info_60[Host_info_60$Resistance == "Susceptible", "Strain"])
```

```
## [1] 26
```

Origin

animal/human/environment/unknown origin

```r
#animal-derived
length(Host_info_60[Host_info_60$Source == "Animal", "Strain"])
```

```
## [1] 28
```

```r
#human-associated
length(Host_info_60[Host_info_60$Source == "Human", "Strain"])
```

```
## [1] 9
```

```r
#environment-associated
length(Host_info_60[Host_info_60$Source == "Environment", "Strain"])
```

```
## [1] 17
```

```r
#unknown origin
length(Host_info_60[Host_info_60$Source == "Unknown", "Strain"])
```

```
## [1] 6
```

Infections / host

```r
# number of infections per host
colSums(HR_60) %>% summary()
```

```
##    Min. 1st Qu.  Median    Mean 3rd Qu.    Max.
##    1.00    4.00   13.00   18.92   31.50   65.00
```

```r
#sd
colSums(HR_60) %>% sd()
```

```
## [1] 17.48539
```

```r
#n
length(colSums(HR_60))
```

```
## [1] 60
```

Phages

Successful infections / phage

```r
# average and max number of infections (strains)
summary(rowSums(HR_60))
```

```
##    Min. 1st Qu.  Median    Mean 3rd Qu.    Max.
##    1.00    9.00   12.00   12.07   15.00   32.00
```

```r
#sd
sd(rowSums(HR_60))
```

```
## [1] 5.354607
```

```r
#n
length(rowSums(HR_60))
```

```
## [1] 94
```

Phages infecting resistant hosts

```r
connected_to_r <- c()

for (phage in phages) {
  n <- neighbors(net, v = phage, mode = "all")
```

```
  res_pattern <- filter(Host_info, Strain %in% names(n)) %>% select(Resistance)
  if("Resistant" %in% res_pattern$Resistance){
    connected_to_r <- append(connected_to_r, phage)
  }
}

length(connected_to_r) # number of phages that infect resistant strains
```

```
## [1] 94
```

```
# all phages infect at least one resistant host
```

Network initialization

```
# initialize network
net_60 <- graph_from_incidence_matrix(HR_60)

# remove the 5 non infected species also as factor levels
Host_info_60$Species <- as.factor(as.character(Host_info_60$Species))

# add source, resistance_phenotype and number of resistances as vertex attributes
for (i in 1:length(Host_info_60$Strain)){
  vertex_attr(net, 'Source', index = as.character(Host_info_60$Strain[i]) ) <-
    as.character(Host_info_60$Source[i])
  vertex_attr(net, 'Resistance_phenotype', index = as.character(Host_info_60$Strain[i]) ) <-
    as.character(Host_info_60$MDR[i])
  vertex_attr(net, 'number_resistances', index = as.character(Host_info_60$Strain[i]) ) <-
    Host_info_60$Number_of_r[i]
  vertex_attr(net, 'Species', index = as.character(Host_info_60$Strain[i]) ) <-
    as.character(Host_info_60$Species[i])
}
#get.vertex.attribute(net)
```

Degree

Whole network

```
#mean
mean(degree(net_60, mode="all"))
```

```
## [1] 14.74026
```

```
#sd
sd(degree(net_60, mode="all"))
```

```
## [1] 12.1051
```

```
#n
length(V(net_60)) # n
```

```
## [1] 154
```

Phages

the degree of a phage refers to the number of strains it infects

```r
#mean
mean(degree(net_60)[names(degree(net_60)) %in% phages])
```

```
## [1] 12.07447
```

```r
#sd
sd(degree(net_60)[names(degree(net_60)) %in% phages])
```

```
## [1] 5.354607
```

```r
#n
length(degree(net_60)[names(degree(net_60)) %in% phages])
```

```
## [1] 94
```

```r
#histogram degree phages
# hist(degree(net_60)[names(degree(net_60)) %in% phages], breaks = 20,
#      main = "Degree Distribution Phages")
dd <-as.data.frame(degree(net_60))
dd_phages <- filter(dd, rownames(dd) %in% phages)
hist_deg_phages <- ggplot(dd_phages, aes(`degree(net_60)`))+
  geom_histogram(breaks = seq(0, 32, 2))+
  xlab("Number of infections")+
  ylab("Number of phages") +
  scale_x_continuous(breaks=seq(0,32,5))

hist_deg_phages
```

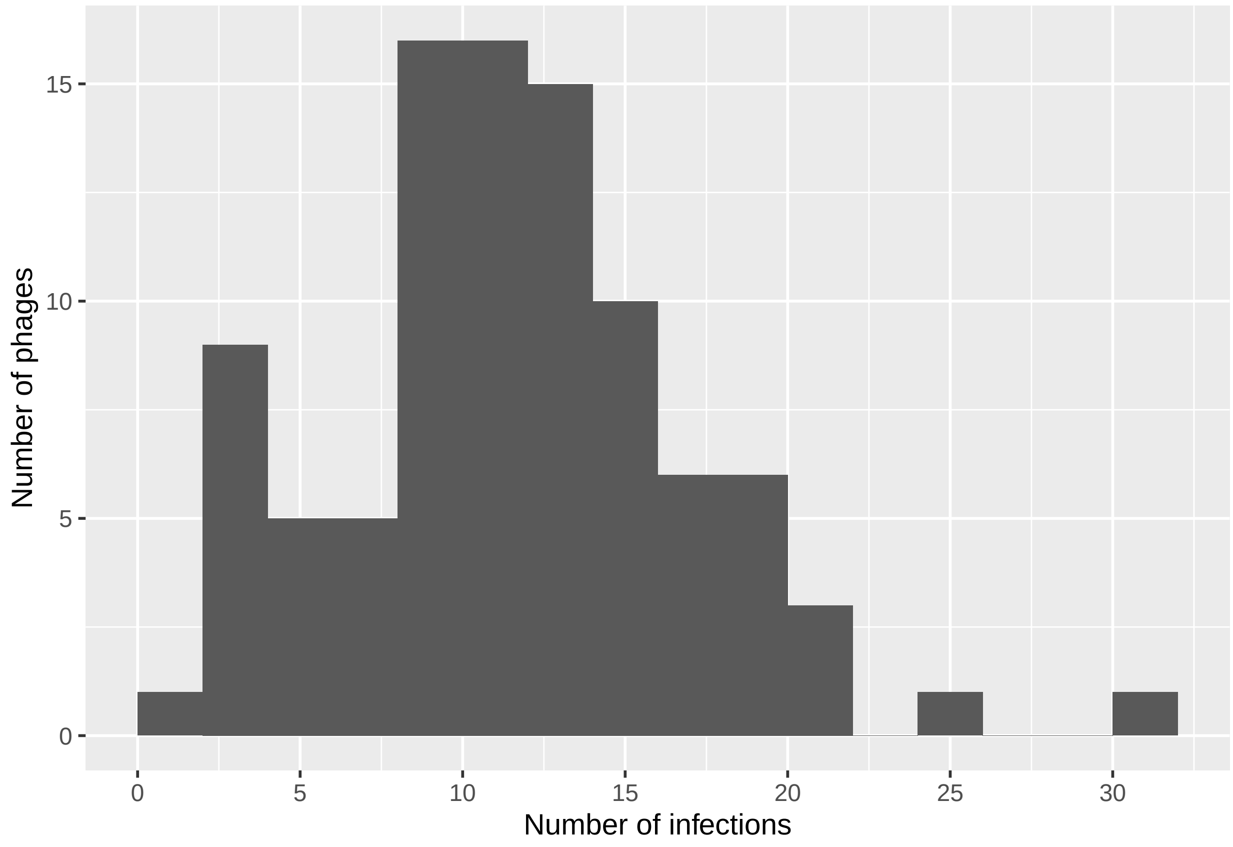

```
#svg(filename="hist_degreePhages.svg")
#hist_deg_phages
#dev.off()
```

Hosts

The degree of a host refers to the number of phages that infect that host

```
# mean degree hosts
mean((degree(net_60)[names(degree(net_60)) %in% hosts_60]))
```

```
## [1] 18.91667
```

```
#sd
sd((degree(net_60)[names(degree(net_60)) %in% hosts_60]))
```

```
## [1] 17.48539
```

```
#n
length((degree(net_60)[names(degree(net_60)) %in% hosts_60]))
```

```
## [1] 60
```

```
#number of hosts that are infected by <= 2 phages
length(degree(net_60)[degree(net_60) <=2])
```

```
## [1] 12
```

```
#histogram degree hosts

# hist(degree(net_60)[names(degree(net_60)) %in% hosts_60], breaks = 25,
#      main = "Degree Distribution Hosts", xlab = "Degree")

dd_hosts <- filter(dd, rownames(dd) %in% hosts_60)
hist_deg_hosts <- ggplot(dd_hosts, aes(`degree(net_60)`))+
  geom_histogram(breaks = seq(0, 32, 2))+
  xlab("Number of infections")+
  ylab("Number of hosts") +
  scale_x_continuous(breaks=seq(0,32,5))

hist_deg_hosts
```

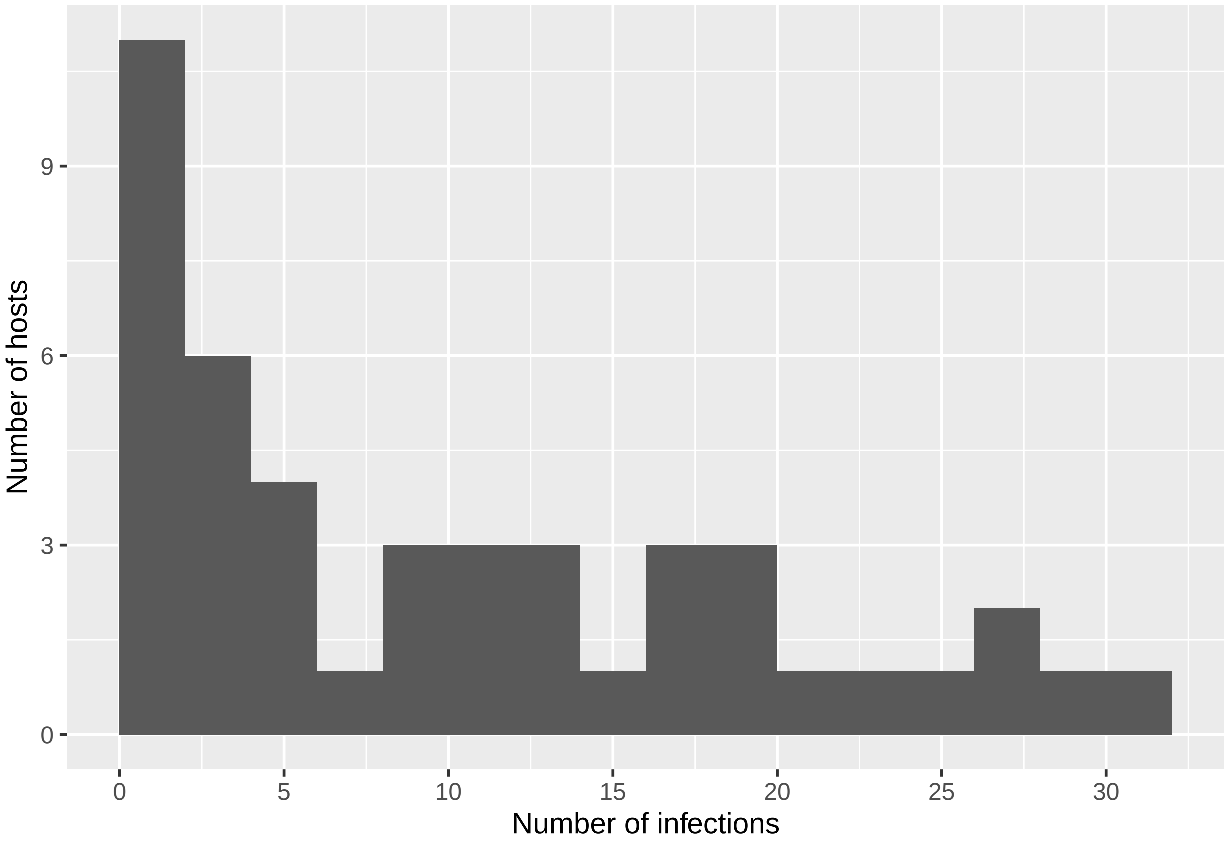


```
#most phage susceptible hosts
degree(net_60)[degree(net_60)>50] #host name and number of infections on that host
```

```
##   S414  I0564 I0507   C5817  I3823
##     56     58    65     53     63
```

```
#characteristics of the most susceptible hosts
Host_info[Host_info$Strain %in% names(degree(net_60)[degree(net_60)>50]),]
```

```
##              Species Coagulase Strain      Source  Resistance     Cluster
## 9   S. epidermidis      CONS   S414     Unknown Susceptible Epidermidis
## 11  S. epidermidis      CONS  I0564      Animal Susceptible Epidermidis
## 36  S. lugdunensis      CONS I0507       Animal   Resistant        <NA>
## 57    S. vitulinus      CONS  C5817 Environment Susceptible      Sciuri
## 117  S. schleiferi      COPS  I3823      Animal Susceptible Intermedius
##            Species_Group
## 9   Epidermidis-Aureus
## 11  Epidermidis-Aureus
## 36                <NA>
## 57              Sciuri
## 117 Hyicus-Intermedius
```

```
#Which hosts are infected by one single phage?
degree(net_60)[degree(net_60)==1]
```

```
## PG-2021_6     I0042     I0838     C3931    C6595      C1890     2638A     C5829
##         1         1         1         1         1         1         1         1
##     C2915
##         1
```

```r
#note that PG-2021_6 is a phage (the only phage that infects only one single host)
```

```r
#characteristics of these hosts
Host_info[Host_info$Strain %in% names(degree(net_60)[degree(net_60)==1]),]
```

```
##                 Species Coagulase Strain      Source  Resistance
## 27           S. hominis      CONS  I0042      Animal Susceptible
## 37        S. nepalensis      CONS  I0838      Animal   Resistant
## 83            S. aureus      COPS  C3931       Human   Resistant
## 87            S. aureus      COPS C6595       Animal   Resistant
## 93            S. aureus      COPS  C1890      Animal   Resistant
## 108 S. pseudintermedius      COPS  2638A     Unknown Susceptible
## 109 S. pseudintermedius      COPS  C5829 Environment   Resistant
## 111 S. pseudintermedius      COPS  C2915       Human   Resistant
##                Cluster      Species_Group
## 27        Haemolyticus Epidermidis-Aureus
## 37  Cohnii-nepalensis      Saprophyticus
## 83           S. aureus Epidermidis-Aureus
## 87                <NA>               <NA>
## 93           S. aureus Epidermidis-Aureus
## 108       Intermedius Hyicus-Intermedius
## 109       Intermedius Hyicus-Intermedius
## 111       Intermedius Hyicus-Intermedius
```

```r
#What phages are infecting these hosts?
specific <- Host_info[Host_info$Strain %in% names(degree(net_60)[degree(net_60)==1]),]$Strain
for (s in specific) {
  print("Host:")
  print(s)
  print("infecting Phage:")
  print(neighbors(net_60, s, mode = "all"))
}
```

```
## [1] "Host:"
## [1] "I0042"
## [1] "infecting Phage:"
## + 1/154 vertex, named, from f5622e7:
## [1] PG-2021_63
## [1] "Host:"
## [1] "I0838"
## [1] "infecting Phage:"
## + 1/154 vertex, named, from f5622e7:
## [1] PG-2021_75
## [1] "Host:"
## [1] "C3931"
## [1] "infecting Phage:"
## + 1/154 vertex, named, from f5622e7:
## [1] PG-2021_47
## [1] "Host:"
## [1] "C6595 "
## [1] "infecting Phage:"
## + 1/154 vertex, named, from f5622e7:
## [1] PG-2021_47
## [1] "Host:"
## [1] "C1890"
```

```
## [1] "infecting Phage:"
## + 1/154 vertex, named, from f5622e7:
## [1] PG-2021_17
## [1] "Host:"
## [1] "2638A"
## [1] "infecting Phage:"
## + 1/154 vertex, named, from f5622e7:
## [1] PG-2021_17
## [1] "Host:"
## [1] "C5829"
## [1] "infecting Phage:"
## + 1/154 vertex, named, from f5622e7:
## [1] PG-2021_17
## [1] "Host:"
## [1] "C2915"
## [1] "infecting Phage:"
## + 1/154 vertex, named, from f5622e7:
## [1] PG-2021_17
```

```
#write.csv(Host_info[Host_info$Strain %in% names(degree(net_60)[degree(net_60)==1]),], file = "specific
```

Node degree by coagulase type

CoNS

```
#mean
mean((degree(net_60)[names(degree(net_60)) %in% CONS$Strain]))
```

```
## [1] 20.59184
```

```
#sd
sd((degree(net_60)[names(degree(net_60)) %in% CONS$Strain]))
```

```
## [1] 16.79772
```

CoPS

```
#mean
mean((degree(net_60)[names(degree(net_60)) %in% COPS$Strain]))
```

```
## [1] 11.45455
```

```
#sd
sd((degree(net_60)[names(degree(net_60)) %in% COPS$Strain]))
```

```
## [1] 19.3513
```

Node degree by species

```
species_info <- data.frame("Species", "n", "mean_degree", "sd_degree",
                           stringsAsFactors = F)
species <- unique(Host_info_60$Species)
for (s in species) {
  hosts <- as.character(Host_info_60[Host_info_60$Species == s, "Strain"])
  species_info <- rbind(species_info, c(s, length(hosts), mean(degree(net_60, hosts)),
                                        sd(degree(net_60, hosts))))
  }
```

```r
species_info <- row_to_names(species_info,1)
species_info
```

```
##                 Species n      mean_degree        sd_degree
## 2           S. arlettae 1                3             <NA>
## 3        S. auricularis 1                6             <NA>
## 4            S. capitis 1               44             <NA>
## 5     S. caprae/capitis 1               13             <NA>
## 6        S. chromogenes 1               33             <NA>
## 7             S. cohnii 1               29             <NA>
## 8        S. epidermidis 7 36.4285714285714 17.5200565339379
## 9            S. equorum 1               35             <NA>
## 10         S. fleuretti 1               13             <NA>
## 11      S. haemolyticus 2                8 5.65685424949238
## 12           S. hominis 1                1             <NA>
## 13            S. hyicus 1               10             <NA>
## 14           S. kloosii 1               24             <NA>
## 15            S. lentus 3 8.66666666666667 7.23417813807024
## 16       S. lugdunensis 1               65             <NA>
## 17        S. nepalensis 1                1             <NA>
## 18          S. pasteuri 1                4             <NA>
## 19      S. pettenkoferi 1               19             <NA>
## 20    S. saprophyticus 1                2             <NA>
## 21            S. sciuri 6               16 9.07744457432817
## 22          S. succinus 2               35 2.82842712474619
## 23         S. vitulinus 3 28.3333333333333 21.5715862498179
## 24           S. warneri 1               15             <NA>
## 25           S. xylosus 9               16 14.4395290781937
## 26            S. aureus 7 8.57142857142857 10.8144524111201
## 27 S. pseudintermedius 3                1                0
## 28        S. schleiferi 1               63             <NA>
```

```r
#species with the highest degree
species_info%>% filter(mean_degree==max(mean_degree))
```

```
##     Species n      mean_degree        sd_degree
## 1 S. lentus 3 8.66666666666667 7.23417813807024
```

Node degree by resistance pattern

```r
resistance_phenotype_info <- data.frame("Resistance phenotype", "n", "mean_degree",
                                        "sd_degree",  stringsAsFactors = F)
res_pattern <- unique(Host_info_60$Resistance)
for (r in res_pattern) {
  hosts <- as.character(Host_info_60[Host_info_60$Resistance == r, "Strain"])
  resistance_phenotype_info <- rbind(resistance_phenotype_info,
                                     c(r, length(hosts), mean(degree(net_60, hosts)),
                                       sd(degree(net_60, hosts))))
  }
resistance_phenotype_info <- row_to_names(resistance_phenotype_info,1)
resistance_phenotype_info
```

```
##   Resistance phenotype  n      mean_degree        sd_degree
## 2            Resistant 34 14.8529411764706  15.884302052448
## 3          Susceptible 26 24.2307692307692 18.3484226947336
```

```r
#table with how many infections on a host of a specific resistance phenotype

resistance_infection <- data.frame(matrix(ncol = 2, nrow = 60))
colnames(resistance_infection) <- c("# infections", "Resistance Phenotype")
rownames(resistance_infection) <- Host_info_60$Strain
for (host in Host_info_60$Strain){
  resistance_infection[host,"# infections"] <- degree(net_60, host)
  resistance_infection[host, "Resistance Phenotype"] <-
    as.character(Host_info_60[Host_info_60$Strain == host,]$Resistance)
}

#write.csv(resistance_infection, "Data_supplfig5.csv")
```

Node degree by source

```r
source_info <- data.frame("Source", "n", "mean_degree", "sd_degree",
                          stringsAsFactors = F)
source <- unique(Host_info$Source)
for (s in source) {
  hosts <- as.character(Host_info_60[Host_info_60$Source == s, "Strain"])
  source_info <- rbind(source_info,
                       c(s, length(hosts), mean(degree(net_60, hosts)),
                         sd(degree(net_60, hosts))))
  }
source_info <- row_to_names(source_info,1)
source_info
```

```
##        Source  n      mean_degree        sd_degree
## 2      Animal 28 18.3928571428571 18.9058863765373
## 3 Environment 17 24.5882352941176 15.1906995540422
## 4     Unknown  6 26.8333333333333 19.0096466739039
## 5       Human  9 4.55555555555556  3.5746017649212
```

Network diameter

the network diameter is the shortest possible path that connects the two most distant node (number of edges)

```r
diameter(net_60, directed = F, weights = NA)
```

```
## [1] 6
```

```r
# nodes along the longest geodesic path
get_diameter(net_60, directed = F)
```

```
## + 7/154 vertices, named, from f5622e7:
## [1] JW205      PG-2021_11 I2468      PG-2021_9  NCC100655  PG-2021_75 I0838
```

Correlation between resistance phenotype and number of phage infections

Test if there is a difference between number of infections between resistant and susceptible hosts

```r
susceptible_hosts <- Host_info_60[Host_info_60$Resistance == 'Susceptible', "Strain"]
resistant_hosts <- Host_info_60[Host_info_60$Resistance == 'Resistant', "Strain"]

deg <- degree(net_60)
```

```r
deg_susceptible <- data.frame('number_of_infections' =
                              deg[names(deg) %in% susceptible_hosts],
                             'resistance_phenotype' =
                              rep('susceptible', length(susceptible_hosts)))

deg_resistant <-data.frame('number_of_infections' =
                            deg[names(deg) %in% resistant_hosts],
                           'resistance_phenotype' =
                            rep('resistant', length(resistant_hosts)))

deg_sr <- rbind(deg_susceptible, deg_resistant)

# compute summary statistics by group

degree_res_pattern_sr <- deg_sr %>% group_by(resistance_phenotype) %>% summarise(
    count = n(),
    mean_infections = mean(number_of_infections, na.rm = TRUE),
    sd = sd(number_of_infections, na.rm = TRUE))
degree_res_pattern_sr
```

```
## # A tibble: 2 x 4
##   resistance_phenotype count mean_infections    sd
##   <fct>                <int>           <dbl> <dbl>
## 1 susceptible             26            24.2  18.3
## 2 resistant               34            14.9  15.9
```

```r
# test data for normality
shapiro.test(deg_susceptible$number_of_infections)
```

```
##
##  Shapiro-Wilk normality test
##
## data:  deg_susceptible$number_of_infections
## W = 0.91676, p-value = 0.03777
```

```r
shapiro.test(deg_resistant$number_of_infections)
```

```
##
##  Shapiro-Wilk normality test
##
## data:  deg_resistant$number_of_infections
## W = 0.82862, p-value = 9.648e-05
```

```r
shapiro.test(deg_sr$number_of_infections)
```

```
##
##  Shapiro-Wilk normality test
##
## data:  deg_sr$number_of_infections
## W = 0.87971, p-value = 2.589e-05
```

```r
wilcox.test(number_of_infections ~ resistance_phenotype, data = deg_sr, exact = FALSE)
```

```
##
##  Wilcoxon rank sum test with continuity correction
##
## data:  number_of_infections by resistance_phenotype
```

```
## W = 591, p-value = 0.02648
## alternative hypothesis: true location shift is not equal to 0
```

Bipartite projections

```
# generate and plot bipartite projections for the two-mode network:
# co-memberships are calculated using igraph's bipartite.projection() function

net2.bp <- bipartite.projection(net_60)
# multiplicity  = TRUE,
# igraph keeps the multiplicity of the edges as an edge attribute called 'weight'.

phage.bp <- net2.bp$proj1
host.bp <- net2.bp$proj2
# since only phages connecting 2 hosts are included,
# we now have only 94 phages in the network
# (phage 14 infects only one host and is thus excluded)
```

Functions

```
# function to get all neighbours from a host and return their seleted attribute
get.neighbours.attribute <- function(host, attribute)
{
  neighbours <-names(neighbors(host.bp, host, mode = 'all'))
  a <-Host_info_60[Host_info_60$Strain %in% neighbours, attribute] %>% as.character()
  return(a)
}

# function which returns the desired attribute of a host
get.host.attribute <- function(host, attribute){
  a <-Host_info_60[Host_info_60$Strain == host, attribute] %>% as.character()
  return(a)
}
```

Host network

hosts: nodes, phages: edges edges are weighed according to the number of shared phages between 2 hosts

```
host.bp
```

```
## IGRAPH 69c2e78 UNW- 60 1030 --
## + attr: name (v/c), weight (e/n)
## + edges from 69c2e78 (vertex names):
##  [1] I4042 --C6866      I4042 --C2709      I4042 --C5847      I4042 --I0507
##  [5] I4042 --I0029      I4042 --C6852      I4042 --C5817      I4042 --I2595
##  [9] I4042 --JW4341     I4042 --Twort      I4042 --I3823      I4042 --I2468
## [13] I4042 --I0564      I4042 --I0042      I4042 --I4644      I4042 --C5816
## [17] I4042 --M1997-2/10 I4042 --3A         I2040B--C6866      I2040B--C5799
## [21] I2040B--S414       I2040B--C5824      I2040B--MP01       I2040B--I2998
## [25] I2040B--I0507      I2040B--C6852      I2040B--I4644      I2040B--C5817
## [29] I2040B--M1997-2/10 I2040B--I2595      I2040B--3A         I2040B--I3823
## + ... omitted several edges
```

Diameter host network

how many phages are needed to connect the two most distant hosts in the network

```
diameter(host.bp, directed = F, weights = NA)
```

```
## [1] 3
```

```
#longest phath through the host network
get_diameter(host.bp, directed = F, weights = NA)
```

```
## + 4/60 vertices, named, from 69c2e78:
## [1] JW205     C5799     NCC100655 I0838
```

Connectance

```
# number of edges in the network
gsize(host.bp)
```

```
## [1] 1030
```

```
# number of nodes in the network
length(V(host.bp))
```

```
## [1] 60
```

```
# connectance: edges/possible number of edges
gsize(host.bp)/(length(V(host.bp))*(length(V(host.bp))-1)/2)
```

```
## [1] 0.5819209
```

Degree

```
#mean
mean(degree(host.bp))
```

```
## [1] 34.33333
```

```
#sd
sd(degree(host.bp))
```

```
## [1] 13.55674
```

```
#n
length(degree(host.bp))
```

```
## [1] 60
```

Degree by species

```
species_info_bp <- data.frame("Species", "n", "mean_degree", "sd_degree",
                              stringsAsFactors = F)
for (s in species) {
  hosts <- as.character(Host_info_60[Host_info_60$Species == s, "Strain"])
  species_info_bp <- rbind(species_info_bp,
                           c(s, length(hosts), mean(degree(host.bp, hosts)),
                                               sd(degree(host.bp, hosts))))
  }
species_info_bp <- row_to_names(species_info_bp,1)
```

```
#dim(species_info_bp)
#head(species_info)
```

```
# species with highest degree
species_info_bp%>% filter(mean_degree==max(mean_degree))
```

```
##         Species n mean_degree sd_degree
## 1 S. schleiferi 1          55      <NA>
```

```
#View(degree_species)
```

Degree by resistance pattern

```
resistance_phenotype_info_bp <- data.frame("Resistance phenotype", "n", "mean_degree",
                                           "sd_degree",  stringsAsFactors = F)
for (r in res_pattern) {
  hosts <- as.character(Host_info_60[Host_info_60$Resistance == r, "Strain"])
  resistance_phenotype_info_bp <- rbind(resistance_phenotype_info_bp,
                                        c(r, length(hosts),
                                          mean(degree(host.bp,
                                                      hosts)),
                                          sd(degree(host.bp, hosts))))
  }
resistance_phenotype_info_bp <- row_to_names(resistance_phenotype_info_bp, 1)
#dim(resistance_phenotype_info)
resistance_phenotype_info_bp
```

```
##   Resistance phenotype  n      mean_degree        sd_degree
## 2            Resistant 34 29.8529411764706 13.8850963330563
## 3          Susceptible 26 40.1923076923077  10.781536924833
```

Degree by source

```
source_info_bp <- data.frame("Source", "n", "mean_degree", "sd_degree",
                             stringsAsFactors = F)
for (s in source) {
  hosts <- as.character(Host_info_60[Host_info_60$Source == s, "Strain"])
  source_info_bp <- rbind(source_info_bp,
                          c(s, length(hosts), mean(degree(host.bp, hosts)),
                                            sd(degree(host.bp, hosts))))
  }
source_info_bp <- row_to_names(source_info_bp,1)
#dim(source_info_bp)
source_info_bp
```

```
##        Source  n      mean_degree        sd_degree
## 2      Animal 28 33.5357142857143 14.4567865464175
## 3 Environment 17 39.8823529411765 11.6451833011613
## 4     Unknown  6 37.8333333333333 12.6081983909941
## 5       Human  9               24 9.08295106229247
```

Neighbours

Neighbours = average number of connected hosts (same as a host's degree)

Neighbors of a particular species

to investibgate to how many other species a particular species is connected to, and to how many hosts of the same species

```
neighbour_info_species <- data.frame("Host", "Species", "n_Neighbours",
                                     "Neighbours_same_Species",
                                     "Neighbours_other_Species",
                                     "n_other_species_connected_to",
                                     stringsAsFactors = F)
hosts_bp <- V(host.bp)
for (host in hosts_60) {
  h_sp <- get.host.attribute(host, "Species")
  n_sp <- get.neighbours.attribute(host, "Species")
  n <- length(get.neighbours.attribute(host, "Species"))
  same <- length(n_sp[n_sp %in% h_sp])
  different_sp <-length(n_sp[!(n_sp %in% h_sp)])
  n_different_sp <- length(unique(n_sp[!(n_sp %in% h_sp)]))
  neighbour_info_species <- rbind(neighbour_info_species,
                                  c(host, h_sp, n, same,
                                    different_sp, n_different_sp))
}

neighbour_info_species <- row_to_names(neighbour_info_species, 1)
#View(neighbour_info)
neighbour_info_species$Neighbours_other_Species <-
  as.numeric(as.character(neighbour_info_species$Neighbours_other_Species))
neighbour_info_species$Neighbours_same_Species <-
  as.numeric(as.character(neighbour_info_species$Neighbours_same_Species))
neighbour_info_species$n_other_species_connected_to <-
  as.numeric(as.character(neighbour_info_species$n_other_species_connected_to))
neighbour_info_species$n_Neighbours <-
  as.numeric(as.character(neighbour_info_species$n_Neighbours))
```

Mean number of other species a host is connected to

```
#mean
mean(neighbour_info_species$n_other_species_connected_to)
```

```
## [1] 17.43333
```

```
# Note: this is the number of different species a host is connected to and
# not the number of hosts of a different species
```

```
#sd
sd(neighbour_info_species$n_other_species_connected_to)
```

```
## [1] 5.224994
```

```
#n
length(neighbour_info_species$n_other_species_connected_to)
```

```
## [1] 60
```

Mean number of host of another species a host is connected to

```
#mean
mean(neighbour_info_species$Neighbours_other_Species)
```

```
## [1] 31.5
```

```
#sd
sd(neighbour_info_species$Neighbours_other_Species)
```

```
## [1] 13.44354
```

```
#n
length(neighbour_info_species$Neighbours_other_Species)
```

```
## [1] 60
```

Neighbours of the same species (number of hosts)

```
#mean
mean(neighbour_info_species$Neighbours_same_Species)
```

```
## [1] 2.833333
```

```
#sd
sd(neighbour_info_species$Neighbours_same_Species)
```

```
## [1] 2.906257
```

```
#n
length(neighbour_info_species$Neighbours_same_Species) #n
```

```
## [1] 60
```

Focus on individual species

```
# focusing on single species
species_neighbour <-neighbour_info_species %>% group_by(Species) %>% summarise(
  n = length(n_Neighbours),
  mean_n_neighbours = mean(n_Neighbours),
  mean_n_neighbours_same_species = mean(Neighbours_same_Species),
  sd_n_neighbours_same_species = sd(Neighbours_same_Species),
  mean_n_neighbours_different_species = mean(Neighbours_other_Species),
  sd_n_neighbours_different_species = sd(Neighbours_other_Species),
  mean_species_connected = mean(n_other_species_connected_to),
  sd_n_species_connected = sd(n_other_species_connected_to))
#species_neighbour
species_info_bp <- left_join(species_info_bp, species_neighbour, by = "Species")
#species_info
```

Number of neighbours of a particular species

```
# number of neighbours of a particular species

neighbours_species <- data.frame("host_species", "neighbour_species",
                                 "mean_n_neighbours", "sd_n_neighbours",
                                 stringsAsFactors = FALSE)

for (species_from in species){ #from
  hosts_species <- Host_info_60 %>% filter(Species == species_from)
  for (species_to in species){ #to
    #print(c(species_from, species_to))
```

```
    n_neighbours <- c()
    for (host in hosts_species$Strain){

      n <- neighbors(host.bp, v = as.character(host))$name %in%
        Host_info_60[Host_info_60$Species == species_to, "Strain"] %>%
        sum()
      n_neighbours <- append(n_neighbours, n)
    }
    #print(mean(n_neighbours))
    #neighbours_species[species_from, species_to] <- mean(n_neighbours)
    neighbours_species <- rbind(neighbours_species, c(species_from, species_to,
                                                      mean(n_neighbours),
                                                      sd(n_neighbours)))
 }
}

neighbours_species <- row_to_names(neighbours_species, 1)
```

Neighbours of a specific environment

```
neighbour_info_env <- data.frame("Host", "Source", "n_Neighbours",
                                 "Neighbours_same_Source",
                                 "Neighbours_other_Source",
                                 "n_other_sources_connected_to",
                                 "Animal_neighbours",
                                 "Human_neighbours",
                                 "Environmental_neighbours",
                                 "Unknown_neighbours",
                                 stringsAsFactors = F)
hosts_bp <- V(host.bp)
for (host in hosts_60) {
  h_sp <- get.host.attribute(host, "Source")
  n_sp <- get.neighbours.attribute(host, "Source")
  n <- length(get.neighbours.attribute(host, "Source"))
  same <- length(n_sp[n_sp %in% h_sp])
  different_sp <-length(n_sp[!(n_sp %in% h_sp)])
  n_different_sp <- length(unique(n_sp[!(n_sp %in% h_sp)]))
  animal <- sum(n_sp == "Animal")
  human <- sum(n_sp == "Human")
  environment <- sum(n_sp == "Environment")
  unknown <- sum(n_sp == "Unknown")
  neighbour_info_env <- rbind(neighbour_info_env,
                              c(host, h_sp, n, same, different_sp,
                                n_different_sp, animal, human,
                                environment, unknown))
}

neighbour_info_env <- row_to_names(neighbour_info_env, 1)
#View(neighbour_info)
neighbour_info_env$Neighbours_other_Source <-
  as.numeric(as.character(neighbour_info_env$Neighbours_other_Source))
neighbour_info_env$Neighbours_same_Source <-
  as.numeric(as.character(neighbour_info_env$Neighbours_same_Source))
```

```r
neighbour_info_env$n_other_sources_connected_to <-
  as.numeric(as.character(neighbour_info_env$n_other_sources_connected_to))
neighbour_info_env$n_Neighbours <-
  as.numeric(as.character(neighbour_info_env$n_Neighbours))

neighbour_info_env$Animal_neighbours <-
  as.numeric(as.character(neighbour_info_env$Animal_neighbours))
neighbour_info_env$Environmental_neighbours <-
  as.numeric(as.character(neighbour_info_env$Environmental_neighbours))
neighbour_info_env$Human_neighbours <-
  as.numeric(as.character(neighbour_info_env$Human_neighbours))
neighbour_info_env$Unknown_neighbours <-
  as.numeric(as.character(neighbour_info_env$Unknown_neighbours))
#dim(neighbour_info_env)
```

Mean number of other environments a host is connected to

```r
# mean
mean(neighbour_info_env$n_other_sources_connected_to)
```

```
## [1] 2.916667
```

```r
# Note: this is the number of different environments a host is connected to
# and not the number of hosts of a different environemnts
```

```r
#sd
sd(neighbour_info_env$n_other_sources_connected_to)
```

```
## [1] 0.2787178
```

```r
#n
length(neighbour_info_env$n_other_sources_connected_to) #n
```

```
## [1] 60
```

Mean number of host of another environemnt a host is connected to

```r
#mean
mean(neighbour_info_env$Neighbours_other_Source)
```

```
## [1] 23.13333
```

```r
#sd
sd(neighbour_info_env$Neighbours_other_Source)
```

```
## [1] 9.900294
```

```r
#n
length(neighbour_info_env$Neighbours_other_Source)
```

```
## [1] 60
```

Neighbours of the same environment

```r
#mean
mean(neighbour_info_env$Neighbours_same_Source)
```

```
## [1] 11.2
```

```r
length(neighbour_info_env$Neighbours_same_Source) #n
```

```
## [1] 60
```

```r
#sd
sd(neighbour_info_env$Neighbours_same_Source)
```

```
## [1] 7.034539
```

```r
#n
sd(neighbour_info_env$Neighbours_same_Source)
```

```
## [1] 7.034539
```

```r
source_neighbour <-neighbour_info_env %>% group_by(Source) %>% summarise(
  n = length(n_Neighbours),
  mean_n_neighbours = mean(n_Neighbours),
  sd_n_neighbours = sd(n_Neighbours),
  mean_n_neighbours_same_source = mean(Neighbours_same_Source),
  sd_n_neighbours_same_source = sd(Neighbours_same_Source),
  mean_n_neighbours_different_source = mean(Neighbours_other_Source),
  sd_n_neighbours_different_source = sd(Neighbours_other_Source),
  mean_environments_connected = mean(n_other_sources_connected_to),
  sd_n_environments_connected = sd(n_other_sources_connected_to),
  mean_animal_neighbours = mean(Animal_neighbours),
  sd_animal_neighbours = sd(Animal_neighbours),
  mean_environmental_neighbours = mean(Environmental_neighbours),
  sd_environmental_neighbours = sd(Environmental_neighbours),
  mean_human_neighbours = mean(Human_neighbours),
  sd_human_neighbours = sd(Human_neighbours),
  mean_unknown_neighbours = mean(Unknown_neighbours),
  sd_unknown_neighbours = sd(Unknown_neighbours))
#source_neighbour
```

Neighbours of a specific resistance phenotype

```r
neighbour_info_res <- data.frame("Host", "Resistance phenotype", "n_Neighbours",
                                 "Neighbours_same_resistance_pt",
                                 "Neighbours_other_resistance_pt",
                                 "n_other_resistance_pt_connected_to",
                                 stringsAsFactors = F)

for (host in hosts_60) {
  h_sp <- get.host.attribute(host, "Resistance")
  n_sp <- get.neighbours.attribute(host, "Resistance")
  n <- length(get.neighbours.attribute(host, "Resistance"))
  same <- length(n_sp[n_sp %in% h_sp])
  different_sp <-length(n_sp[!(n_sp %in% h_sp)])
  n_different_sp <- length(unique(n_sp[!(n_sp %in% h_sp)]))
  neighbour_info_res <- rbind(neighbour_info_res,
                              c(host, h_sp, n, same,
                                different_sp, n_different_sp))
}

neighbour_info_res <- row_to_names(neighbour_info_res, 1)
#View(neighbour_info)
```

```r
neighbour_info_res$Neighbours_other_resistance_pt <-
  as.numeric(as.character(neighbour_info_res$Neighbours_other_resistance_pt))
neighbour_info_res$Neighbours_same_resistance_pt <-
  as.numeric(as.character(neighbour_info_res$Neighbours_same_resistance_pt))
neighbour_info_res$n_other_resistance_pt_connected_to <-
  as.numeric(as.character(neighbour_info_res$n_other_resistance_pt_connected_to))
neighbour_info_res$n_Neighbours <-
  as.numeric(as.character(neighbour_info_res$n_Neighbours))
#dim(neighbour_info_res)
```

Mean number of other resistance phenotype a host is connected to

```r
#mean
mean(neighbour_info_res$n_other_resistance_pt_connected_to)
```

```
## [1] 1
```

```r
# all susceptible hosts are connected to at least one resistant host
```

```r
#sd
sd(neighbour_info_res$n_other_resistance_pt_connected_to)
```

```
## [1] 0
```

```r
#n
length(neighbour_info_res$n_other_resistance_pt_connected_to)
```

```
## [1] 60
```

Mean number of host of another resistance phenotype a host is connected to

```r
#mean
mean(neighbour_info_res$Neighbours_other_resistance_pt)
```

```
## [1] 17.63333
```

```r
#sd
sd(neighbour_info_res$Neighbours_other_resistance_pt)
```

```
## [1] 7.070988
```

```r
#n
length(neighbour_info_res$Neighbours_other_resistance_pt)
```

```
## [1] 60
```

Neighbours of the same resisstance phenotype

```r
# mean
mean(neighbour_info_res$Neighbours_same_resistance_pt)
```

```
## [1] 16.7
```

```r
#sd
sd(neighbour_info_res$Neighbours_same_resistance_pt)
```

```
## [1] 6.85516
```

```r
#n
length(neighbour_info_res$Neighbours_same_resistance_pt)
```

```
## [1] 60
```

```r
rpt_neighbour <-neighbour_info_res %>% group_by(`Resistance phenotype`) %>% summarise(
  n = length (n_Neighbours),
  mean_n_neighbours = mean(n_Neighbours),
  mean_n_neighbours_same_resistance_pt = mean(Neighbours_same_resistance_pt),
  sd_n_neighbours_same_resistance_pt = sd(Neighbours_same_resistance_pt),
  mean_n_neighbours_different_resistance_pt = mean(Neighbours_other_resistance_pt),
  sd_n_neighbours_different_resistance_pt = sd(Neighbours_other_resistance_pt),
  mean_resistance_pt_connected = mean(n_other_resistance_pt_connected_to),
  sd_n_resistance_pt_connected = sd(n_other_resistance_pt_connected_to))
rpt_neighbour
```

```
## # A tibble: 2 x 9
##   `Resistance phe~     n mean_n_neighbou~ mean_n_neighbou~ sd_n_neighbours~
##   <chr>            <int>            <dbl>            <dbl>            <dbl>
## 1 Resistant           34             29.9             14.3             7.47
## 2 Susceptible         26             40.2             19.8             4.37
## # ... with 4 more variables: mean_n_neighbours_different_resistance_pt <dbl>,
## #   sd_n_neighbours_different_resistance_pt <dbl>,
## #   mean_resistance_pt_connected <dbl>, sd_n_resistance_pt_connected <dbl>
```

```r
resistance_phenotype_info_bp <- left_join(resistance_phenotype_info_bp, rpt_neighbour,
                                          by = "Resistance phenotype")
```

Number of susceptible bacteria a resistant host is connected to

```r
#average number of susceptible neighbours of a resistant host in the network
mean(neighbour_info_res[neighbour_info_res$`Resistance phenotype`== "Resistant", "Neighbours_other_resi
```

```
## [1] 15.55882
```

```r
#maximum
max(neighbour_info_res[neighbour_info_res$`Resistance phenotype`== "Resistant", "Neighbours_other_resis
```

```
## [1] 26
```

```r
neighbour_info_res[neighbour_info_res$Neighbours_other_resistance_pt == max(neighbour_info_res[neighbou
```

```
##      Host Resistance phenotype n_Neighbours Neighbours_same_resistance_pt
## 7   I2468            Resistant           52                            26
## 10   S414          Susceptible           50                            24
## 25 I0507             Resistant           53                            27
##    Neighbours_other_resistance_pt n_other_resistance_pt_connected_to
## 7                              26                                  1
## 10                             26                                  1
## 25                             26                                  1
```

```r
#histogram
hist_sneighbours <- ggplot(data = neighbour_info_res[neighbour_info_res$`Resistance phenotype`== "Resis
  geom_histogram(breaks = seq(0, 28, by = 2))+
  xlab("Number of susceptible neighbours")+
  ylab("Number of resistant hosts")+
  #geom_vline(xintercept = 16, colour = "red")+
  scale_x_continuous(breaks=seq(0,28,5))

#svg(filename="hist_sNeighbours.svg")
#hist_sneighbours
```

```
#dev.off()

hist_sneighbours
```

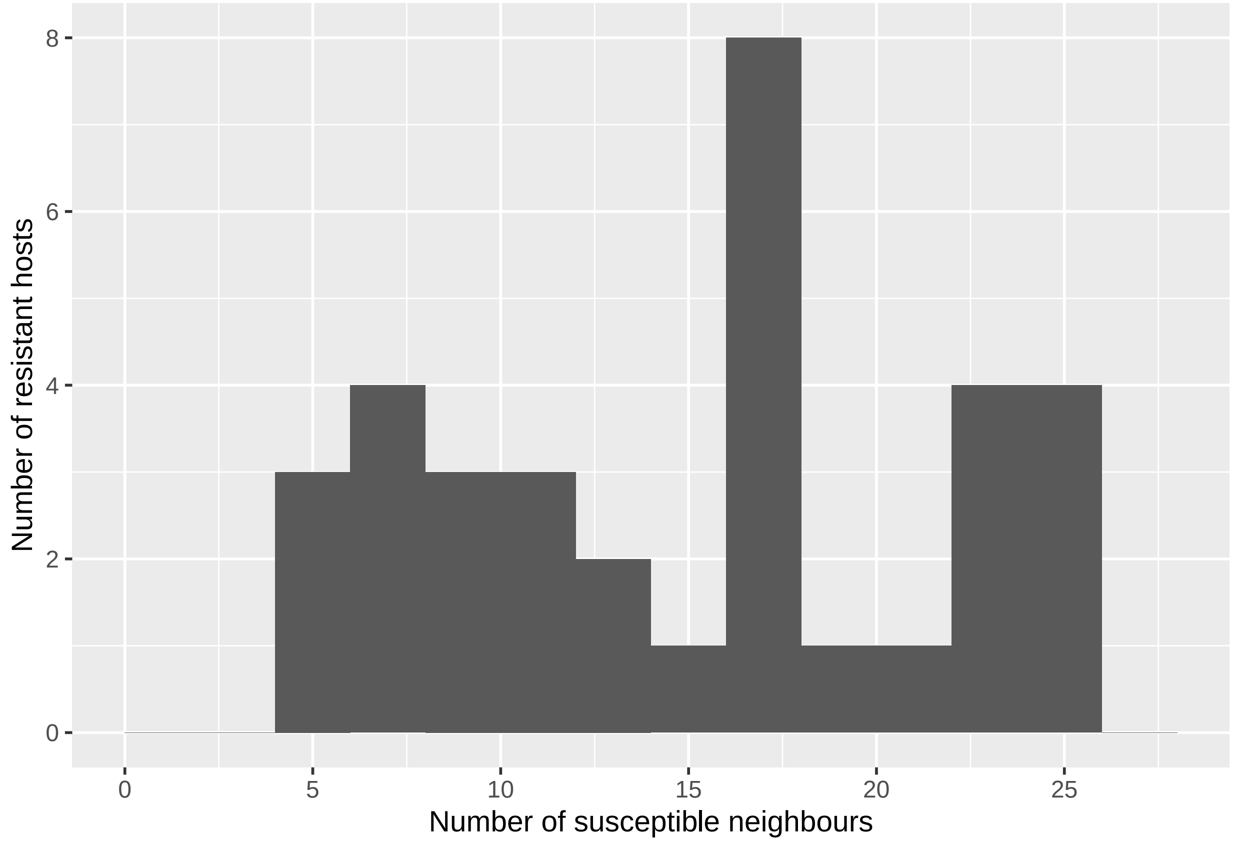

Shared phages

Shared phages are represented as weight of the edge between two nodes.

```
# maximal number of phages connecting 2 hosts:
max(E(host.bp)$weight)
```

```
## [1] 58
```

```
#length(E(host.bp)$weight)#n edges
```

```
# best connected hosts:
E(host.bp)[E(host.bp)$weight == 58] #best connected hosts
```

```
## + 1/1030 edge from 69c2e78 (vertex names):
## [1] I0507 --I3823
```

```
# hosts connected by over 50 phages:
E(host.bp)[E(host.bp)$weight > 50] #hosts connected by over 50 phages
```

```
## + 4/1030 edges from 69c2e78 (vertex names):
## [1] S414  --I0564  I0564 --I0507  I0564 --I3823  I0507 --I3823
```

```
# to calculate the average number of shared phages between 2 hosts,
# we need to include the hosts that aren't connected at all
host_am <-as_adj(net2.bp$proj2, sparse = F)
```

```r
# adjacency matrix host network: 0 = not connected(no shared phages),
# 1 = at least 1 shared phage
# E(host.bp)$weight #number of shared phages
```

```r
# number of hosts that aren't connected:
sum(host_am == 0)/2 -30 # -30 to exclude self interactions (60/2)
```

```
## [1] 740
```

```r
#sum(host_am != 0)/2 # number of edges
# total possible interactions: 1770 (60*59/2)
```

```r
# matrix with number of shared phages between hosts
shared_phages <- matrix(nrow = 60, ncol = 60)
colnames(shared_phages) <- Host_info_60$Strain
rownames(shared_phages) <- Host_info_60$Strain


for (host in hosts_bp$name){
  for (neighbour in hosts_bp$name){ #all neighbours
    connecting_phages<-host.bp[host][neighbour] # number of connecting phages
    shared_phages[host, neighbour] <- connecting_phages

  }
}

# write.csv(shared_phages, "shared_phages_species.csv")
```

Average number of connecting phages

```r
# include hosts that aren't connected ( = connected by 0 phages)
connecting_phages <- c(E(host.bp)$weight,replicate(740, 0))
# 1770 possible interactions, 1030 real interactions
# 740 hosts that aren't connected at all
```

```r
# average number of connecting phages
mean(connecting_phages)
```

```
## [1] 4.303955
```

```r
#sd
sd(connecting_phages)
```

```
## [1] 7.879049
```

```r
#n
length(connecting_phages)
```

```
## [1] 1770
```

Between species

```r
# initialize dataframe
df_species <- data.frame('Host', 'Host_species', 'Neighbour', 'Neighbour_species',
                         'Connecting_phages',stringsAsFactors = FALSE)

# loop through all hosts
```

```r
for (host in hosts_bp$name){
  s<-as.character(get.host.attribute(host, "Species")) #host species
  for (neighbour in hosts_bp$name){ #all neighbours
    if(neighbour != host){
      ns<-as.character(get.host.attribute(neighbour, "Species"))# neighbor source
      connecting_phages<-host.bp[host][neighbour] # number of connecting phages
      r <- c(as.character(host), s, as.character(neighbour), ns, connecting_phages)
      df_species<-rbind(df_species,r)

    }
  }
}


df_species <- row_to_names(df_species, row_number = 1)
#head(df_species)
#dim(df_species) # 60*59, each host occurs once as host and one time as neighbour

df_species$Connecting_phages<-as.numeric(as.character(df_species$Connecting_phages))
#head(df_species)
```

Average number of phages shared between species

```r
connecting_phages_between <- df_species %>% filter(Host_species != Neighbour_species)
```

```r
#mean
mean(connecting_phages_between$Connecting_phages)
```

```
## [1] 4.056423
```

```r
#sd
sd(connecting_phages_between$Connecting_phages)
```

```
## [1] 7.541368
```

```r
#n
dim(connecting_phages_between) # number of interactions between species = 3332
```

```
## [1] 3332    5
```

Average number of phages shared within species

```r
# shared phages within species:
connecting_phages_within <- df_species %>% filter(Host_species == Neighbour_species)
#dim(connecting_phages_within)
```

```r
#mean
mean(connecting_phages_within$Connecting_phages)
```

```
## [1] 8.269231
```

```r
#sd
sd(connecting_phages_within$Connecting_phages)
```

```
## [1] 11.36163
```

```r
#n
dim(connecting_phages_within) # 208 interactions within species
```

```
## [1] 208   5
```

Is there a significant difference between shared phages within / between species?

```r
# test for normality
shapiro.test(connecting_phages_between$Connecting_phages)
```

```
##
##  Shapiro-Wilk normality test
##
## data:  connecting_phages_between$Connecting_phages
## W = 0.59472, p-value < 2.2e-16
```

```r
shapiro.test(connecting_phages_within$Connecting_phages)
```

```
##
##  Shapiro-Wilk normality test
##
## data:  connecting_phages_within$Connecting_phages
## W = 0.73084, p-value < 2.2e-16
```

```r
# test wheter there is a significant difference in the number of
# shared phages between/within species:
wilcox.test(connecting_phages_between$Connecting_phages,
            connecting_phages_within$Connecting_phages)
```

```
##
##  Wilcoxon rank sum test with continuity correction
##
## data:  connecting_phages_between$Connecting_phages and connecting_phages_within$Connecting_phages
## W = 237492, p-value = 1.93e-15
## alternative hypothesis: true location shift is not equal to 0
```

```r
# shared phages between different species:
phages_species <-df_species %>% group_by(Host_species, Neighbour_species) %>% summarise(
  mean_connecting_phages = mean(Connecting_phages),
  sd_connecting_phages = sd(Connecting_phages))
#phages_species

#dim(phages_species)
#sum(phages_species$Host_species == phages_species$Neighbor_species)
#number of same species interactions
#(the other  18 species have only one member and thus aren't in the ds)
```

Shared phages between environments

dataframe in which we have each possible connection(hosts), their environments and connecting phages in a row, dimensions of the df: 60*59 since we are not interested in the host forming connections with itself note that this df is somewhat redundant, as the host pair xy will once occur as host x neighbour y and then host y, neighbour x

```r
# initialize dataframe
df_env <- data.frame('Host', 'Host_source', 'Neighbour', 'Neighbour_source',
                     'Connecting_phages',stringsAsFactors = FALSE)

hosts_bp <- V(host.bp)$name
# loop through all hosts in the bp network
```

```r
for (host in hosts_bp){
  s<-as.character(get.host.attribute(host, "Source")) # host source
  for (neighbour in hosts_bp){ # all neighbours
    if(neighbour != host){ # no self-interactions
      ns<-as.character(get.host.attribute(neighbour, "Source")) # neighbor source
      connecting_phages<-host.bp[host][neighbour] #shared phages
      df_env<-rbind(df_env,c(as.character(host), s, as.character(neighbour),
                             ns, connecting_phages))

    }
  }
}


df_env <- row_to_names(df_env, row_number = 1)
#head(df_env)
#rows: number of interactions between strains
#dim(df_env)/2

df_env$Host<-as.factor(df_env$Host)
df_env$Host_source<-as.factor(df_env$Host_source)
df_env$Neighbour<-as.factor(df_env$Neighbour)
df_env$Neighbour_source<-as.factor(df_env$Neighbour_source)
df_env$Connecting_phages<-as.numeric(as.character(df_env$Connecting_phages))

# add additional column indicating if it is between or within environments
df <- mutate(df_env, within_env = ifelse(Host_source == Neighbour_source,
                                          'within', 'between'))
```

```r
# mean connecting phages by environments
df_connecting_phages_env <- df %>% group_by(Host_source, Neighbour_source) %>%
  summarise(
  n = length (Connecting_phages),
  mean_connecting_phages=mean(Connecting_phages),
  sd_connecting_phages = sd(Connecting_phages))
#df_connecting_phages_env
#sum(df_connecting_phages_env$n)/2
#double the number of possible interactions because of the redundancy discussed above
```

note: here I assess how many shared phages any 2 hosts from 2 specific environments share -> 2 host that aren't connected are added as 0 sharing phages and the mean is calculated from all possible interactions.Also each interaction between environments is listed twice

Shared phages within and between environment

```r
# how many phages connect hosts within / between environments on average?
df %>% group_by(within_env) %>% summarise(
  mean_connecting_phages = mean(Connecting_phages),
  sd_connecting_phages = sd(Connecting_phages),
  n = length(Connecting_phages)/2)
```

```
## # A tibble: 2 x 4
##   within_env mean_connecting_phages sd_connecting_phages     n
##   <chr>                       <dbl>                <dbl> <dbl>
## 1 between                      4.12                 7.63  1205
```

```
## 2 within                              4.69                 8.38   565
```

```
# length/2 as each interaction is listed twice
```

Is there a significant difference between shared phages within/between environment?

```
# test for normality
shapiro.test(df$Connecting_phages)
```

```
##
##  Shapiro-Wilk normality test
##
## data:  df$Connecting_phages
## W = 0.60228, p-value < 2.2e-16
```

```
# not normal distributed
```

the p-value is > 0.05% which indicates that there is no difference between and within environments.

```
# difference between the different environments?
wilcox.test(df$Connecting_phages ~ df$within_env)
```

```
##
##  Wilcoxon rank sum test with continuity correction
##
## data:  df$Connecting_phages by df$within_env
## W = 1315504, p-value = 0.08981
## alternative hypothesis: true location shift is not equal to 0
```

Shared phages between resistance phenotypes

```
# initialize dataframe
df_rpt <- data.frame('Host', 'Host_resistance_pt', 'Neighbour', 'Neighbour_resistance_pt',

                                 'Connecting_phages',stringsAsFactors = FALSE)
#loop through all hosts
for (host in hosts_bp){
  s<-as.character(get.host.attribute(host, "Resistance")) # host source
  for (neighbour in hosts_bp){ # all neighbours
    if(neighbour != host){ #no self-interactions
      ns<-as.character(get.host.attribute(neighbour, "Resistance")) # neighbor source
      connecting_phages<-host.bp[host][neighbour]
      df_rpt<-rbind(df_rpt,c(as.character(host), s, as.character(neighbour),
                             ns, connecting_phages))

    }
  }
}


df_rpt <- row_to_names(df_rpt, row_number = 1)
#dim(df_rpt)/2
#again all possible interactions are listed twice

df_rpt$Host<-as.factor(df_rpt$Host)
df_rpt$Host_resistance_pt<-as.factor(df_rpt$Host_resistance_pt)
df_rpt$Neighbour<-as.factor(df_rpt$Neighbour)
```

```r
df_rpt$Neighbour_resistance_pt<-as.factor(df_rpt$Neighbour_resistance_pt)
df_rpt$Connecting_phages<-as.numeric(as.character(df_rpt$Connecting_phages))

# add additional column indicating if it is between or within resistance group
df_rpt <- mutate(df_rpt, within_rpt = ifelse(Host_resistance_pt ==
                                              Neighbour_resistance_pt, 'within', 'between'))
```

```r
# mean connecting phages by resistance phenotype
df_connecting_phages_rpt <- df_rpt %>%
  group_by(Host_resistance_pt, Neighbour_resistance_pt) %>%
  summarise(n = length(Connecting_phages),
  mean_connecting_phages = mean(Connecting_phages),
  sd_connecting_phages = sd(Connecting_phages),
  n = length(Connecting_phages))

# remove duplicated interactions
df_connecting_phages_rpt <- df_connecting_phages_rpt[c(1:2,4),]
df_connecting_phages_rpt[df_connecting_phages_rpt$Host_resistance_pt ==
                           df_connecting_phages_rpt$Neighbour_resistance_pt, ]$n <-
  df_connecting_phages_rpt[df_connecting_phages_rpt$Host_resistance_pt ==
                             df_connecting_phages_rpt$Neighbour_resistance_pt, ]$n/2
df_connecting_phages_rpt
```

```
## # A tibble: 3 x 5
## # Groups:   Host_resistance_pt [2]
##   Host_resistance_~ Neighbour_resista~     n mean_connecting_~ sd_connecting_ph~
##   <fct>             <fct>              <dbl>             <dbl>             <dbl>
## 1 Resistant         Resistant            561              2.54              5.64
## 2 Resistant         Susceptible          884              4.35              8.00
## 3 Susceptible       Susceptible          325              7.23              9.78
```

```r
# how many phages connect hosts within / between resistance group on average?
df_rpt %>% group_by(within_rpt) %>% summarise(
  n = length (Connecting_phages),
  mean_connecting_phages = mean(Connecting_phages),
  sd_connecting_phages = sd(Connecting_phages),
  n = length(Connecting_phages)/2)
```

```
## # A tibble: 2 x 4
##   within_rpt     n mean_connecting_phages sd_connecting_phages
##   <chr>      <dbl>                  <dbl>                <dbl>
## 1 between      884                   4.35                 7.99
## 2 within       886                   4.26                 7.76
```

Is there a difference in shared phages within/between resistance groups?

```r
# see if there's a difference between the number of connecting phages
# between and within resistance group
shapiro.test(df_rpt$Connecting_phages)
```

```
##
##  Shapiro-Wilk normality test
##
## data:  df_rpt$Connecting_phages
## W = 0.60228, p-value < 2.2e-16
```

```r
# not normal distributed
```

```r
# difference between the different resistance groups?
wilcox.test(df_rpt$Connecting_phages ~ df_rpt$within_rpt)
```

```
##
##  Wilcoxon rank sum test with continuity correction
##
## data:  df_rpt$Connecting_phages by df_rpt$within_rpt
## W = 1584550, p-value = 0.535
## alternative hypothesis: true location shift is not equal to 0
```

Shared phages between cluster groups

```r
# initialize dataframe
df_sg <- data.frame('Host', 'Host_species_group', 'Neighbour', 'Neighbour_species_group',

                                 'Connecting_phages',stringsAsFactors = FALSE)
#loop through all hosts
for (host in hosts_bp){
  s<-as.character(get.host.attribute(host, "Species_Group")) # host source
  for (neighbour in hosts_bp){ # all neighbours
    if(neighbour != host){ #no self-interactions
      ns<-as.character(get.host.attribute(neighbour, "Species_Group")) # neighbor source
      connecting_phages<-host.bp[host][neighbour]
      df_sg<-rbind(df_sg,c(as.character(host), s, as.character(neighbour),
                             ns, connecting_phages))

    }
  }
}


df_sg <- row_to_names(df_sg, row_number = 1)
#dim(df_rpt)/2
#again all possible interactions are listed twice

df_sg$Host<-as.factor(df_sg$Host)
df_sg$Host_species_group<-as.factor(df_sg$Host_species_group)
df_sg$Neighbour<-as.factor(df_sg$Neighbour)
df_sg$Neighbour_species_group<-as.factor(df_sg$Neighbour_species_group)
df_sg$Connecting_phages<-as.numeric(as.character(df_sg$Connecting_phages))

# add additional column indicating if it is between or within species group
df_sg <- mutate(df_sg, within_sg = ifelse(Host_species_group ==
                                            Neighbour_species_group, 'within', 'between'))
```

Mean connecting phages by species group

```r
df_connecting_phages_sg <- df_sg %>%
  group_by(Host_species_group, Neighbour_species_group) %>%
  summarise(n = length(Connecting_phages),
  mean_connecting_phages = mean(Connecting_phages),
  sd_connecting_phages = sd(Connecting_phages),
  n = length(Connecting_phages))
```

```
## Warning: Factor `Host_species_group` contains implicit NA, consider using
## `forcats::fct_explicit_na`

## Warning: Factor `Neighbour_species_group` contains implicit NA, consider using
## `forcats::fct_explicit_na`
```

```
df_connecting_phages_sg
```

```
## # A tibble: 35 x 5
## # Groups:   Host_species_group [6]
##    Host_species_gro~ Neighbour_species~     n mean_connecting_~ sd_connecting_p~
##    <fct>             <fct>              <int>             <dbl>            <dbl>
##  1 Auricularis       Epidermidis-Aureus    19              1.42             2.27
##  2 Auricularis       Hyicus-Intermedius     6              3.5              2.74
##  3 Auricularis       Saprophyticus         13              3.31             2.59
##  4 Auricularis       Sciuri                10              0.7              1.89
##  5 Auricularis       <NA>                  11              2                2.61
##  6 Epidermidis-Aure~ Auricularis           19              1.42             2.27
##  7 Epidermidis-Aure~ Epidermidis-Aureus   342              5.37             9.75
##  8 Epidermidis-Aure~ Hyicus-Intermedius   114              4.48             9.80
##  9 Epidermidis-Aure~ Saprophyticus        247              4.24             6.82
## 10 Epidermidis-Aure~ Sciuri               190              2.87             6.18
## # ... with 25 more rows
```

```
sum(df_connecting_phages_sg$n)/2
```

```
## [1] 1770
```

```
#write.csv(df_connecting_phages_sg, file= "Shared_phages_species_groups.csv")
```

How many phages connect hosts within / between species group on average?

```
df_sg %>% group_by(within_sg) %>% summarise(
  n = length (Connecting_phages),
  mean_connecting_phages = mean(Connecting_phages),
  sd_connecting_phages = sd(Connecting_phages),
  n = length(Connecting_phages)/2)
```

```
## # A tibble: 3 x 4
##   within_sg     n mean_connecting_phages sd_connecting_phages
##   <chr>     <dbl>                  <dbl>                <dbl>
## 1 between     867                   4.02                 7.50
## 2 within      309                   5.83                 8.95
## 3 <NA>        594                   3.92                 7.74
```

Is there a difference between the number of connecting phages within/between species groups?

```
# see if there's a difference between the number of connecting phages
# between and within resistance group
shapiro.test(df_sg$Connecting_phages)
```

```
##
##  Shapiro-Wilk normality test
##
## data:  df_sg$Connecting_phages
## W = 0.60228, p-value < 2.2e-16
```

```
# not normal distributed
```

```
# difference between the different resistance groups?
wilcox.test(df_sg$Connecting_phages ~ df_sg$within_sg)
```

```
##
##  Wilcoxon rank sum test with continuity correction
##
## data:  df_sg$Connecting_phages by df_sg$within_sg
## W = 462916, p-value = 1.653e-07
## alternative hypothesis: true location shift is not equal to 0
```

Shared phages between specific hosts

Hosts: * S. xylosus I2595 * S. aureus PS187 * S. epidermidis S414 * S. xylosus JW4341 * S. xylosus M1997-2/10 * S. aureus 3A * S. aureus RN4220

```
hosts_wta <- c("I2595", "PS187", "S414", "JW4341", "M1997-2/10", "3A ")
shared_phages <- matrix(0, nrow = length(hosts_wta), ncol = length(hosts_wta))
rownames(shared_phages) <- hosts_wta
colnames(shared_phages) <- hosts_wta
#shared_phages["S414", "3A "] <- 3
for (host1 in hosts_wta){
   for (host2 in hosts_wta){
     shared <- E(host.bp)[host1 %--% host2]$weight
     connected <- get.edge.ids(host.bp, c(host1, host2), directed = TRUE, error = FALSE,
  multi = FALSE)
     if (connected != 0){
       shared_phages[host1, host2] <- shared
     }
   }
 }
print(shared_phages)
```

```
##            I2595 PS187 S414 JW4341 M1997-2/10 3A
## I2595          0     8   26      5         30 18
## PS187          8     0    8      0          8  7
## S414          26     8    0      2         26 17
## JW4341         5     0    2      0          7  1
## M1997-2/10    30     8   26      7          0 19
## 3A            18     7   17      1         19  0
```

```
#write.csv(shared_phages, file = "Shared_phages_wta.csv")
```

Modularity and Nestedness

```r
#package version
sessionInfo()
```

```
## R version 3.6.2 (2019-12-12)
## Platform: x86_64-apple-darwin15.6.0 (64-bit)
## Running under: macOS  10.16
##
## Matrix products: default
## BLAS:   /Library/Frameworks/R.framework/Versions/3.6/Resources/lib/libRblas.0.dylib
## LAPACK: /Library/Frameworks/R.framework/Versions/3.6/Resources/lib/libRlapack.dylib
##
## locale:
## [1] de_CH.UTF-8/de_CH.UTF-8/de_CH.UTF-8/C/de_CH.UTF-8/de_CH.UTF-8
##
## attached base packages:
## [1] stats     graphics  grDevices utils     datasets  methods   base
##
## other attached packages:
## [1] readxl_1.3.1    dplyr_0.8.5     vegan_2.5-6     lattice_0.20-40
## [5] permute_0.9-5   RInSp_1.2.4     lpbrim_1.0.0
##
## loaded via a namespace (and not attached):
##  [1] Rcpp_1.0.4       knitr_1.28       cluster_2.1.0    magrittr_1.5
##  [5] splines_3.6.2    MASS_7.3-51.5    tidyselect_1.0.0 R6_2.4.1
##  [9] rlang_0.4.5      stringr_1.4.0    tools_3.6.2      parallel_3.6.2
## [13] grid_3.6.2       nlme_3.1-145     mgcv_1.8-31      xfun_0.12
## [17] htmltools_0.4.0  assertthat_0.2.1 yaml_2.2.1       digest_0.6.25
## [21] tibble_2.1.3     crayon_1.3.4     Matrix_1.2-18    purrr_0.3.3
## [25] glue_1.3.2       evaluate_0.14    rmarkdown_2.1    stringi_1.4.6
## [29] cellranger_1.1.0 pillar_1.4.3     compiler_3.6.2   pkgconfig_2.0.3
```

Prepare data

```r
#import data: Phage host range matrix
Phage_HR <- read.csv("Data/Phage_HR_binary_woL.csv", row.names=1, stringsAsFactors = F, sep = ";")
Phage_HR <- Phage_HR[1:94,] #get rid of species and coagulase rows, as well as of phage K
#dim(Phage_HR)
```

```r
#remove hosts that aren't infected at all
rn <- rownames(Phage_HR)
Phage_HR <- Phage_HR %>% mutate_all(function(x) as.numeric(as.character(x))) %>%
as.matrix() #turn into numeric matrix
rownames(Phage_HR) <- rn
HR <- Phage_HR[,colSums(Phage_HR) != 0] #only include the hosts that are infected dim(HR)
```

interaction matrix of 94 phages and 60 hosts, 63 hosts aren't infected by any of the phages

Modularity

```
mod <- findModules(HR, iter = 100, sparse = FALSE)
modules <- getmodules(mod) #extract modules
mod$Q #modularity
```

```
## [1] 0.3825923
```

Number of Interactions within Modules

```
interactions <- 0
for (i in 1:length(modules)) {interactions <- interactions + (sum(modules[[i]]))}
print(interactions)
```

```
## [1] 832
```

Significance Testing

```
#function to extract calculate modularity with findModules and extract modularity value
getModularity <- function(m){
  modu <- findModules(m, iter = 100, sparse = FALSE)
  Q <- modu$Q
  return(Q)
}

#compare modularity with 1000 randomly generated matrices that have the same number #of interactions
sim_mod <- oecosimu(HR, getModularity, "r00", nsimul= 100, alternative = "greater")
sim_mod
```

```
## oecosimu object
##
## Call: oecosimu(comm = HR, nestfun = getModularity, method = "r00",
## nsimul = 100, alternative = "greater")
##
## nullmodel method 'r00' with 100 simulations
##
## alternative hypothesis: statistic is greater than simulated values
##
##           statistic    SES    mean     50%    95% Pr(sim.)
## statistic   0.38279 49.857 0.21508 0.21487 0.2207 0.009901 **
## ---
## Signif. codes:  0 '***' 0.001 '**' 0.01 '*' 0.05 '.' 0.1 ' ' 1
```

Nestedness

```
nodf <- nestednodf(HR)
nodf
```

```
## N columns   : 38.0255
## N rows      : 42.0794
## NODF        : 40.91096
## Matrix fill: 0.2012411
```

Significance Testing

```
sim <- oecosimu(HR, nestednodf, "r00", nsimul= 100, alternative = "greater")
sim
```

```
## oecosimu object
##
## Call: oecosimu(comm = HR, nestfun = nestednodf, method = "r00", nsimul
## = 100, alternative = "greater")
##
## nullmodel method 'r00' with 100 simulations
##
## alternative hypothesis: statistic is greater than simulated values
##
## N columns  : 38.0255
## N rows     : 42.0794
## NODF       : 40.91096
## Matrix fill: 0.2012411
##
##           statistic    SES   mean    50%    95% Pr(sim.)
## N.columns    38.026 43.170 21.040 21.064 21.611 0.009901 **
## N.rows       42.079 48.705 21.251 21.266 21.853 0.009901 **
## NODF         40.911 50.443 21.190 21.172 21.735 0.009901 **
## ---
## Signif. codes:  0 '***' 0.001 '**' 0.01 '*' 0.05 '.' 0.1 ' ' 1
```

Species Specificity Phages

Phages are thought to infect specific species, however it seems like many of our phages are able to infect several species.

What we want: look at the number of hits whithin a species, if this is > 50% of the total hits of this phage we define this phage as species specific

Note: The script always has to be run as a whole to avoid errors

```
rm(list =ls())
library(tidyverse)
```

```
## -- Attaching packages ----------------------------------------------------------------------------

## v ggplot2 3.3.0     v purrr   0.3.3
## v tibble  2.1.3     v dplyr   0.8.5
## v tidyr   1.0.2     v stringr 1.4.0
## v readr   1.3.1     v forcats 0.5.0

## -- Conflicts -------------------------------------------------------------------------------------
## x dplyr::filter() masks stats::filter()
## x dplyr::lag()    masks stats::lag()
```

```
#import dataset

#HR_Species <- read.csv("Data/HR_Phages_by_Species.csv", row.names=1)
HR_Species <- read.csv("Data/HR_Phages_by_Species_newn.csv", sep=";", row.names = 1)
#head(HR_Species) #rownames: phages, colnames: species
#View(HR_Species)
phages <- rownames(HR_Species) #note phage K(reference) is still in the dataset
hosts <- colnames(HR_Species)
```

```
#Idea:
#calc RowSum -> new column: total infections
HR_Species['total_infections'] <- rowSums(HR_Species)
HR_Species[,'specificity'] <- 'generalist'
#default: set specificity to generalist -> change later if specific
#head(HR_Species)
```

```
#go through the dataset row by row
for (phage in phages){
  hr <- HR_Species[phage,]
  #print(hr)
  #then go through the entries of this row and calc the percentage
  #of the infections in this strain relative to the total infections
  for (host in hosts){
    infections_strain <- (hr[host])
    #if the value >50% we say the phage is specific for that strain and save it
    #-> new column: strain specificity
    if(infections_strain >= as.numeric(as.character(hr['total_infections']))/2){
       HR_Species[phage, 'specificity'] <- host
```

```r
        HR_Species[phage, 'percent_infections'] <-
          infections_strain/as.numeric(as.character(hr['total_infections']))
        }
    }
}

#View(HR_Species)
```

```r
# write.csv(HR_Species, 'Species_Specificity_Phages.csv')
```

```r
sum(HR_Species$specificity != 'generalist')
```

```
## [1] 35
```

```r
sum(HR_Species$specificity == 'generalist')
```

```
## [1] 60
```
