# Supplementary material for: Multi-species host range of staphylococcal phages isolated from wastewater
Source: Nat Commun. 2021 Nov 29;12:6965. doi: 10.1038/s41467-021-27037-6 (PMC8629997; doi:10.1038/s41467-021-27037-6)
Supplement: Supplementary file 14 — Reporting Summary [file 41467_2021_27037_MOESM14_ESM.pdf]

nature research

Corresponding author(s): Elena Gómez-Sanz

Last updated by author(s): 02/10/2021

Reporting Summary

Nature Research wishes to improve the reproducibility of the work that we publish. This form provides structure for consistency and transparency in reporting. For further information on Nature Research policies, see our Editorial Policies and the Editorial Policy Checklist.

Statistics

For all statistical analyses, confirm that the following items are present in the figure legend, table legend, main text, or Methods section.

n/a	Confirmed	
☐	☒	The exact sample size (n) for each experimental group/condition, given as a discrete number and unit of measurement
☒	☐	A statement on whether measurements were taken from distinct samples or whether the same sample was measured repeatedly
☐	☒	The statistical test(s) used AND whether they are one- or two-sided<br>*Only common tests should be described solely by name; describe more complex techniques in the Methods section.*
☒	☐	A description of all covariates tested
☐	☒	A description of any assumptions or corrections, such as tests of normality and adjustment for multiple comparisons
☐	☒	A full description of the statistical parameters including central tendency (e.g. means) or other basic estimates (e.g. regression coefficient) AND variation (e.g. standard deviation) or associated estimates of uncertainty (e.g. confidence intervals)
☐	☒	For null hypothesis testing, the test statistic (e.g. F, t, r) with confidence intervals, effect sizes, degrees of freedom and P value noted<br>*Give P values as exact values whenever suitable.*
☒	☐	For Bayesian analysis, information on the choice of priors and Markov chain Monte Carlo settings
☒	☐	For hierarchical and complex designs, identification of the appropriate level for tests and full reporting of outcomes
☒	☐	Estimates of effect sizes (e.g. Cohen's d, Pearson's r), indicating how they were calculated

Our web collection on statistics for biologists contains articles on many of the points above.

Software and code

Policy information about availability of computer code

Data collection	No software was used.
Data analysis	Pacbio for phage genome assembly: SMRT Link v8.0.0.80529, vSMRT Tools 8.0.0.80502<br>Server for all bioinformatic work: linux v3.10.0-1127.18.2.el7.x86_64 centos/7.9.2009 with conda v4.6.14 and phyton v3.6.8<br>Illumina read trimming: Bioconda trimmomatic v0.39<br>Illumina phage genome assembly: Bioconda spades v3.14.1<br>Multiphate for phage annotation v.1.0 with python v3.8.2, biopython v1.76, Prodigal v2.6.3, jackhmmer v3.3, blast+ v. 2.9.0<br>tRNA scan: Bioconda tRNA-Scan-SE v2.0.5<br>Phage genome ends: PhageTerm v1.0.12<br>Biopython package v1.68, entrez-direct v13.3 to retrieve staphylococcal phage genomes from GenBank<br>Phage closest relative analysis (Average Nucleotide Identity): JSpecies<br>Phylogenetic analysis: Prodigal (v2.60), DIAMOND (v0.9.14), R (v3.2.5), R libraries: phangorn (v2.5.5)<br>Network analysis: R version 3.6.2 (2019-12-12), Platform: x86_64-apple-darwin15.6.0 (64-bit), Running under: macOS Catalina 10.15.7, with igraph v1.2.5<br>Modularity and nestedness: lpbrim v1.0.0 and vegan v2.5-6 R packages<br>Data manipulation with tidyverse v1.3.0<br>Search for viral sequences: VIBRANT v 1.2.1

For manuscripts utilizing custom algorithms or software that are central to the research but not yet described in published literature, software must be made available to editors and reviewers. We strongly encourage code deposition in a community repository (e.g. GitHub). See the Nature Research guidelines for submitting code & software for further information.

Data

Policy information about availability of data

All manuscripts must include a data availability statement. This statement should provide the following information, where applicable:

- Accession codes, unique identifiers, or web links for publicly available datasets
- A list of figures that have associated raw data
- A description of any restrictions on data availability

All isolated bacteriophages are available upon request. The phage genomes sequenced in this study have been deposited as nucleotide sequences in the GenBank database under the accession codes MZ417315-MZ417354 (see Supplementary Data 7, column H) within Bioproject PRJNA663854 (https://www.ncbi.nlm.nih.gov/bioproject/PRJNA663854). The genome of S. epidermidis strain S414 was deposited in the European Nucleotide Archive under the accession code ERZ3124167 (https://www.ebi.ac.uk/ena/browser/view/PRJEB42698). Source data are provided with this paper.

Field-specific reporting

Please select the one below that is the best fit for your research. If you are not sure, read the appropriate sections before making your selection.

☐ Life sciences ☐ Behavioural & social sciences ☒ Ecological, evolutionary & environmental sciences

For a reference copy of the document with all sections, see nature.com/documents/nr-reporting-summary-flat.pdf

Ecological, evolutionary & environmental sciences study design

All studies must disclose on these points even when the disclosure is negative.

Study description	Host range study of 94 staphylococcal bacteriophages that were isolated from water. Water was collected from the inlet and outlet of a wastewater treatment plant in Zürich, Switzerland. Bacteriophages were isolated and their host range assessed on 119 staphylococcal, 4 macrococcal and two enteroccocus strains. The characterization of the established phage-bacteria interaction matrix is described. Whole genome sequencing analysis and electron microscopy was conducted in a subset of 40 phages. The antibiotic resistance gene encapsidation capacity of a selected group is also disclosed.
Research sample	The sample choice were naturally occurring staphylococcal phages isolated from wastewater and from treated water from a wastewater treatment plant in Zurich, Switzerland. This source was the sampling choice as it is a hotspot for microbial communities from diverse sources (human household waste, animal husbandry, industry, etc) and microbial interactions and potential DNA exchange due to particular conditions (cell density, oxygen level, chemical and other residues, microbial stress, etc). Hence, a dense matrix of bacteria-infecting bacteriophages is also expected. Staphylococci are not predominantly recovered from human or animal waste, yet they are present and can be amplified (both, bacterial cells and extracellular phages) by targeted enrichment steps.
Sampling strategy	Water samples were collected in sterile glassware. Samples (2.5 L each) were taken at the entrance after the mechanical clearance, and from the effluent that is directly released into the lake of Zürich. No further sampling was needed. We determined this amount of water to be sufficient based on former bibliography and scientific evidence, as well as based internal former sampling efforts at the same treatment plant for phages infecting different hosts, of similar expected abundances. Particularly, three rounds of phage enrichment steps were performed using five in-house constituted staphylococcal cocktails, addition of fresh media, overnight incubation and serial membrane filtration steps each round. For the induced phages recovered from staphylococcal isolates in the water samples, staphylococcal enrichment media (TSB+ 6.5% NaCl) was used to increase initial cell counts in the water fraction, followed by prophage induction assays (UV, Mitomycin C) to recover phages from the staphylococcal lysogens. These enrichment steps maximize the chances to recover staphylococcal phages from remarkably lower initial abundance.
Data collection	Characteristics of wastewater treatment plant processing and capacity were provided to Pauline Göller, Viona Bernardi and Elena Gomez-Sanz. Details of all bacterial strains included in the study were collected by Elena Gomez-Sanz and Pauline Göller. Data on the bacterial cocktail constitution were collected by Ekaterina Khatchatourova, Viona Bernardi and Pauline Göller. Generated data from phage isolation and phage clusterization (based on host range assays) were collected and reported by Pauline Göller, Viona Bernardi, Dominic Lorgé, Nesrine Amri, Annika Naumann and Natasa Radulovic. All these data were stored in Excel spreadsheets and electronic lab-journals in word or Latex files. Genomic data were generated and reported by Tabea Elsener, Pauline Göller and Felipe Hernandes Coutinho. Encapsidation data was collected and reported by Tabea Elsener. The later datasets were stored as excel and/or word files, R markdown files, .fasta files and related sequencing data formats. Micrographs were generated and collected by Pauline Göller, and stored as image supported files (.tiff).
Timing and spatial scale	Water samples were taken once on the 24th July 2018. It was estimated a single sampling date (two sampling points, inlet wastewater and outlet treated water, 2.5 L each, ca. 100 meter apart spaced by water treatment compartments) was sufficient to obtain a large collection of staphylococcal phages. There was not sampling restrictions so additional samplings had been taken, in case the initial sampling would have resulted unsuccessful or insufficient.<br>Bacteriophage enrichment and isolation started the same day and performed within 1 week after sampling.
Data exclusions	no data was excluded from the analysis.
Reproducibility	All phage-host infection assays (94 x 123) were done in duplicates. If incongruities occurred, a final third evaluation was performed. All experiments were reproduced with the same conditions to reliably support the conclusions stated in the manuscript. Of note, later infection assays of a subset of phages propagated in different hosts with respect to original experiments revealed not 100% reproducibility in several cases (ca. 5%). This might be due to modifications of the phage genome during infection in the later hosts.

Randomization	There was no requirement for randomization. All samples and data points were analyzed equally with no sub-sampling. Associations and quantifications were performed using computational pipelines applied equally to all.
Blinding	Blinding was not possible as experimental conditions required prior identification of phages and bacterial hosts used. All data points were generated with equal conditions and analyzed equally to all of them and replicates.

Did the study involve field work? ☒ Yes ☐ No

Field work, collection and transport

Field conditions	Water samples were taken from a wastewater treatment plant in Au, Zürich from the inlet (after the mechanical clearance) and outlet, which is directly released into the lake of Zürich. Date: July 24th, 2018. Partially cloudy weather, 25°C.
Location	Abwasserreinigungsanlagen<br>Alte Landstrasse 8<br>8804 Au ZH<br>Switzerland
Access & import/export	Samples were collected by staff working at the wastewater treatment plant into sterile, autoclaved glassware. Samples were sterile filtered and stored at 4 °C within the first hour after collection.
Disturbance	no disturbance.

Reporting for specific materials, systems and methods

We require information from authors about some types of materials, experimental systems and methods used in many studies. Here, indicate whether each material, system or method listed is relevant to your study. If you are not sure if a list item applies to your research, read the appropriate section before selecting a response.

Materials & experimental systems

n/a	Involved in the study	
☒	☐	Antibodies
☒	☐	Eukaryotic cell lines
☒	☐	Palaeontology and archaeology
☒	☐	Animals and other organisms
☒	☐	Human research participants
☒	☐	Clinical data
☒	☐	Dual use research of concern

Methods

n/a	Involved in the study	
☒	☐	ChIP-seq
☒	☐	Flow cytometry
☒	☐	MRI-based neuroimaging
